# Estradiol ameliorates AD pathology and cognitive deficits by SORLA-mediated APP endosomal trafficking

**DOI:** 10.1186/s13195-026-02027-2

**Published:** 2026-03-25

**Authors:** Fengting Cao, Jinxi Liu, Hongchun Zuo, Xulian Lin, Yuhong Su, Chang Wang, Huixian Cui, Juan Du, Yizhou Zhang

**Affiliations:** 1 Department of Human Anatomy, Neuroscience Research Center, Hebei Key Laboratory of Neurodegenerative Disease Mechanism, Hebei Medical University, Shijiazhuang, 050017 China; 2 Department of Pathology, Metabolic Diseases and Cancer Research Center, Hebei Medical University, Shijiazhuang, 050017 China; 3 Beijing Rehabilitation Hospital, Musculoskeletal Rehabilitation Center, Capital Medical University, Beijing, 100144 China

**Keywords:** Alzheimer’s disease, Estradiol, SORLA, APP, ERα, Endosomal trafficking

## Abstract

Alzheimer’s disease (AD) shows a notable female bias in both prevalence and severity. This bias is closely linked to declining estradiol levels during menopause. Our study demonstrated that estradiol can inhibit the accelerated progression of AD caused by ovariectomy (OVX) in female APP/PS1 mice. Transcriptome analysis revealed that estradiol prevented the decline in sortilin-related receptor 1 (*Sorl1*), which encodes the sorting receptor SORLA, in the hippocampus of female APP/PS1-OVX mice. Neuronal *Sorl1* knockdown blocked estradiol’s ability to alleviate AD symptoms. Mechanistically, estradiol enhanced SORLA-mediated redirection of amyloid precursor protein (APP) trafficking from early endosomes toward the Golgi apparatus and lysosomes, which were involved in non-amyloidogenic processing and reducing Aβ levels. Additionally, estradiol promoted estrogen receptor α (ERα) binding to the *Sorl1* promoter, thereby activating SORLA expression. Notably, estradiol ameliorated AD progression in female APP/PS1-OVX mice through ERα, and this effect depended on SORLA. Overall, estradiol mitigated AD pathology by activating *Sorl1* transcription via ERα, which in turn directed APP endosomal trafficking through SORLA. This pathway provides valuable mechanistic insights into sex differences in AD and highlights potential therapeutic targets.

**Supplementary Information:**

The online version contains supplementary material available at 10.1186/s13195-026-02027-2.

Alzheimer’s disease (AD) is a progressive neurodegenerative disorder resulting in cognitive impairment, primarily due to amyloid-β (Aβ) plaques, neurofibrillary tangles, synaptic dysfunction, and neuronal death. Epidemiological studies have confirmed that females are more likely to experience a higher incidence, greater severity, and faster progression of AD than males [1, 2]. Lower estrogen levels during menopause are believes to contribute to sex differences in AD [1, 3], but the specific mechanisms are not yet fully understood.

Estrogen is mainly produced by developing ovarian follicles and has neurotrophic and neuroprotective effects. The decline in estrogen levels, especially estradiol (E2, its main form), during menopause increases the risk of age-related disorders and neurodegeneration [4, 5]. Both in vitro and in vivo studies confirm that E2 protects against AD [6–8]; however, the underlying mechanism is still not fully understood. In the classical pathway, E2 exerts its biological effects through the transcription factor estrogen receptor-α (ERα) and estrogen receptor-β (ERβ). When E2 binds to ERα and ERβ, these receptors recruit coactivators that attach to estrogen response elements on DNA and regulate the transcription of genes involved in proliferation, differentiation, and survival. Besides genomic transcriptional activation, ERα and ERβ may also interact with other transcription factors and act downstream of growth factor signaling [5]. E2 also signals via the G protein-coupled estrogen receptor 1 (GPER1), which activates calcium flux, PLCβ–PKC, ERK1/2, and Rho–ROCK signalling pathways in physiology and disease [9, 10]. ERα, ERβ, and GPER1 are widely distributed in the central nervous system [11]; however, the neuroprotective role and mechanism of E2 in AD require further investigation.

The accumulation of Aβ plaques in the brain is a major pathological hallmark of AD. According to the amyloid hypothesis, Aβ peptides induce hyperphosphorylation and propagation of tau [12, 13], cognitive impairment [14, 15], neurotoxicity, and neuronal cell death [16]. Aβ peptides are generated through the amyloidogenic pathway, wherein amyloid precursor protein (APP) is sequentially cleaved by β-secretase and γ-secretase, producing a soluble amyloid precursor protein β (sAPPβ) fragment and Aβ peptides [17]. In contrast, soluble amyloid precursor protein α (sAPPα) and shorter non-fibrillar fragments are produced via the non-amyloidogenic pathway through the sequential action of α-secretase and γ-secretase [17]. APP is a type of transmembrane glycoprotein that plays important physiological roles in intracellular signal transduction, synapses and neural plasticity, and cell adhesion [18]. After being synthesized in the endoplasmic reticulum, full-length APP is transported to the cell surface through the Golgi and trans-Golgi network (TGN) [19]. An increase in APP delivery or a decrease in its internalization from the cell surface enhances non-amyloidogenic processing, while retaining APP in acidic compartments, such as early endosomes, promotes amyloidogenesis [20–21]. Neuronal activity promotes the gathering of APP and secretases in recycling endosomes, which is an early step in Aβ production that involves various intracellular organelles [21]. β-secretase located on the endosomal membrane cleaves APP into sAPPβ and C-terminal fragments (CTFβ). APP is directed to early endosomes and then sorted into three distinct pathways: (a) some APP molecules are recycled to the cell surface, (b) others are transported retrogradely to the TGN via a retromer-mediated pathway, and (c) some APP molecules are targeted to late endosomes that fuse with lysosomes for degradation [22–24]. Although previous studies have shown that E2 lowers Aβ production in AD models [25, 26], how it affects the sorting pathway of APP and Aβ production is remains unclear.

In this study, we performed bilateral ovariectomy (OVX) on female APP/PS1 mice (APP/PS1-OVX) to reduce E2 levels, because natural aging in rodents does not lower estrogen sufficiently [27]. APP/PS1 mice develop associated Aβ plaques at 6–9 months and cognitive deficits at around 12 months [28]. Notably, these features were accelerated after OVX but can be suppressed by E2 administration. In addition, we found that E2 prevented the decline in the expression of sortilin-related receptor 1 (*Sorl1*) following ovariectomy. Mechanically, E2 promoted sortilin-related receptor A (SORLA, *Sorl1* encoding protein)-mediated APP endosomal trafficking via ERα, thereby reducing Aβ production. These results contributes to a better understanding of the pathophysiology of AD and its sex-specific characteristics.

## Results

### Estradiol alleviates the accelerated progression of AD caused by ovariectomy in female APP/PS1 mice

**Fig. 1:**
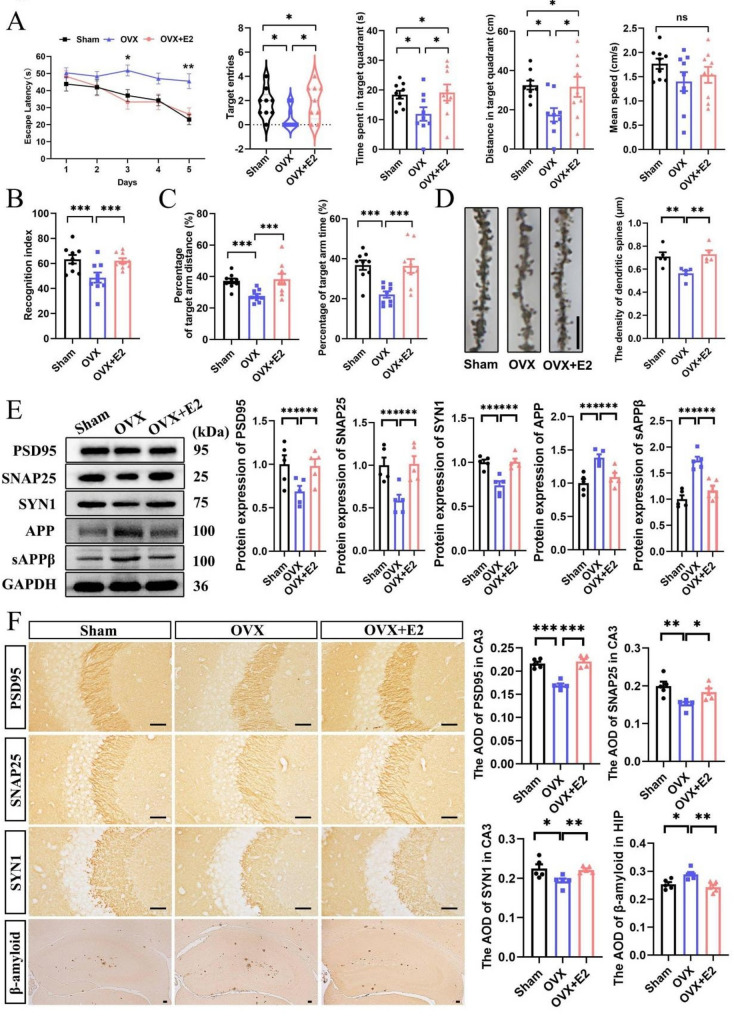
Estradiol alleviates the accelerated progression of AD caused by ovariectomy in female APP/PS1 mice. **A** The latency to find the hidden platform during training trials, as well as the target entries, time spent and distance in the target quadrant, and the mean speed during probe trials in the MWM (*n* = 9). **B** Recognition index in the NOR (*n* = 9). **C** The distance and time spent in the target (novel) arm in the Y maze (*n* = 9). **D** The hippocampal dendritic spine density (bar: 10 μm, *n* = 5). **E** WB for PSD95, SNAP25, SYN1, APP, and sAPPβ expression in the hippocampus (*n* = 5). **F** IHC for PSD95, SNAP25, SYN1 in the hippocampal CA3, and Aβ deposition in the hippocampus (bar: 50 μm, *n* = 5). Sham, female sham APP/PS1 mice; OVX, female ovariectomized APP/PS1 mice; OVX+E2, female ovariectomized APP/PS1 mice with estradiol supplementation. *P < 0.05, **P < 0.01, ***P < 0.001, ns: No significant difference

To clarify the role of E2 in AD progression, we performed OVX to reduce E2 levels in 4-month-old female APP/PS1 mice and then supplemented with E2 through intraperitoneal injection (Fig. S1A). We performed behavioral assessments including the morris water maze (MWM, Fig. 1A; Fig. S1B), novel object recognition (NOR, Fig. 1B), and Y maze (Fig. 1C) tests, and found that OVX exacerbated cognitive deficits in 4-month-old female APP/PS1 mice, but E2 administration reversed these impairments. E2 also prevented the reduction in hippocampal dendritic spine density of female APP/PS1-OVX mice (Fig. 1D). Western blotting (WB, Fig. 1E) and immunohistochemistry (IHC, Fig. 1F; Fig. S1C) results showed that E2 prevented the decline of postsynaptic density protein 95 (PSD95), synaptosomal-associated protein 25 (SNAP25), and synapsin I (SYN1) levels in the hippocampus of female APP/PS1-OVX mice. WB results demonstrated that E2 reduced the elevated expression of APP and its cleaved product sAPPβ, an important product of the amyloidogenic pathway, caused by OVX (Fig. 1E). Additionally, Aβ staining results indicated a marked increase in the hippocampus of female APP/PS1-OVX mice compared to APP/PS1-Sham mice; however, E2 prevented this effect (Fig. 1F). These data indicate that E2 alleviates the accelerated progression of AD in female APP/PS1-OVX mice.

### Estradiol prevents the decline in SORLA level in the hippocampus of APP/PS1-OVX mice

**Fig. 2:**
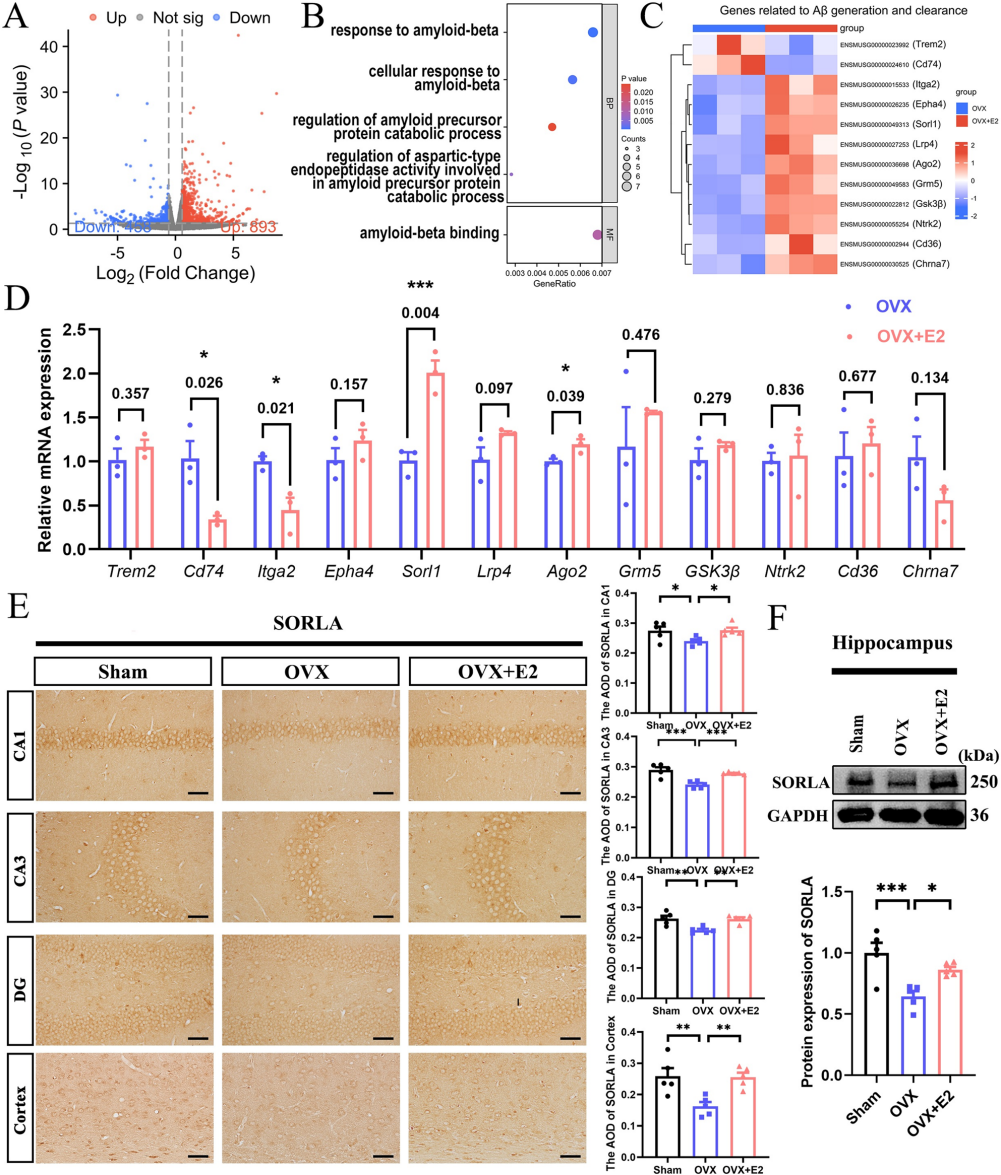
Estradiol prevents the decline in SORLA level in the hippocampus of APP/PS1-OVX mice. **A** Volcano plot showing the DEGs identified by RNA-seq from the hippocampus. **B** GO analysis of Aβ related genes. **C** Heatmap of Aβ related genes. **D** qRT-PCR for relative mRNA levels of the Aβ related genes (*n* = 3). **E** IHC for SORLA expression in the hippocampus and cerebral cortex (bar: 50 μm, *n* = 5). **F** WB for SORLA expression in the hippocampus (*n* = 5). Sham, female sham APP/PS1 mice; OVX, female ovariectomized APP/PS1 mice; OVX+E2, female APP/PS1 mice with estradiol supplementation. *P < 0.05, **P < 0.01, ***P < 0.001

To investigate how E2 affects AD progression, we performed RNA sequencing (RNA-seq) on the hippocampus of APP/PS1-OVX mice treated with vehicle control or E2. We identified 1,361 differentially expressed genes (DEGs), including 893 upregulated and 468 downregulated genes (Fig. 2A). Since E2 reduces Aβ deposition, we screened DEGs involved in Aβ metabolism to explore the molecular mechanisms. The GO enrichment analysis revealed that the key processes associated with Aβ metabolism included “response to amyloid-beta”, “cellular response to amyloid-beta”, “regulation of the amyloid precursor protein catabolic process”, “regulation of aspartic-type endopeptidase activity involved in the amyloid precursor protein catabolic process”, and “amyloid-beta binding” and so on (Fig. 2B). We identified 12 genes associated with Aβ metabolism, including *TREM2*, *Cd74*, *Itga2*, *Epha4*, *Sorl1*, *Lrp4*, *Ago2*, *Grm5*, *Gsk3β*, *Ntrk2*, *Cd36*, and *Chrna7* (Table S1). A heatmap illustrated the levels of these genes in the hippocampus of APP/PS1-OVX mice treated with vehicle control or E2 (Fig. 2C). Quantitative real-time PCR (qRT-PCR) analysis further confirmed the changes in *Cd74* (*P* = 0.026), *Sorl1* (*P* = 0.004), and *Ago2* (*P* = 0.039) expression observed in RNA-seq results. Notably, *Sorl1* exhibited the most significant expression difference (Fig. 2D). SORLA (*Sorl1* encoding protein) acts as a sorting receptor that regulates the endo-lysosomal trafficking of various substrates [29]. IHC and WB results showed that OVX reduced SORLA expression in the hippocampus neurons of female APP/PS1 mice; however, E2 reversed this effect (Fig. 2E, F). In addition, IHC also results showed that E2 prevented the decline in SORLA level in the cerebral cortex of female APP/PS1-OVX mice (Fig. 2E).

### Estradiol mitigates the accelerated progression of AD in female APP/PS1-OVX mice via SORLA

**Fig. 3:**
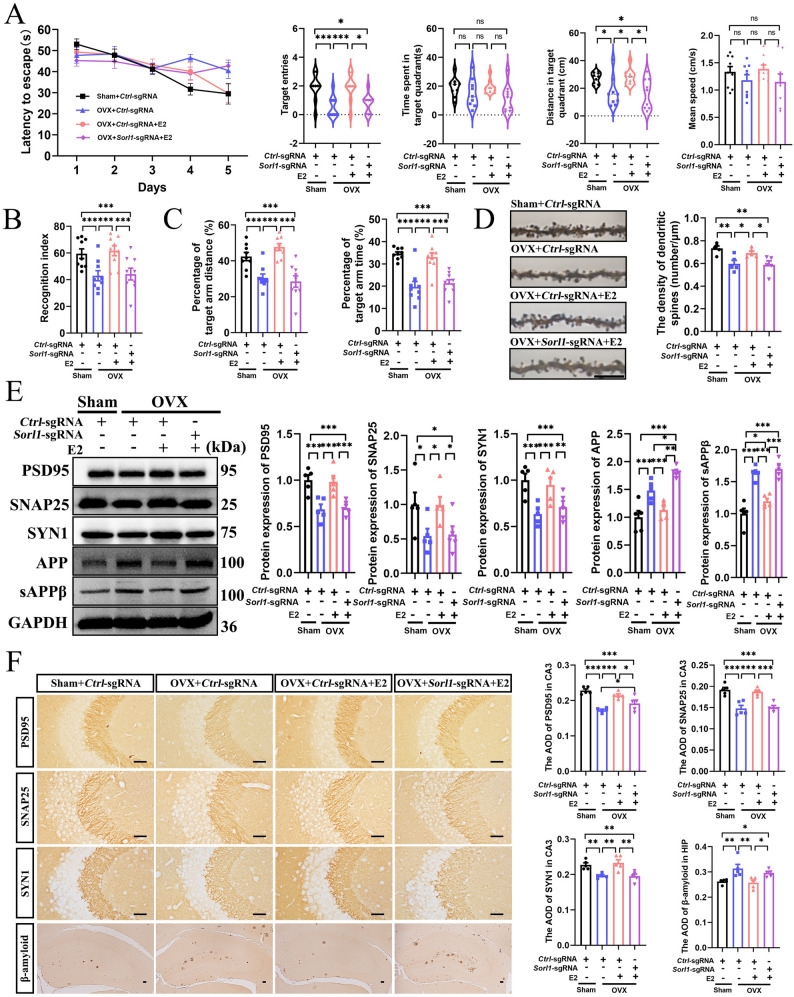
Estradiol mitigates the accelerated progression of AD in female APP/PS1-OVX mice via SORLA. **A** The latency to find the hidden platform during training trials, the target entries, the time spent and distance in the target quadrant, and the mean speed during probe trials in the MWM (*n* = 9). **B** Recognition index in the NOR (*n* = 9). **C** The distance and time spent in the target (novel) arm in the Y maze (*n* = 9). **D** Golgi staining for the hippocampal dendritic spine density (bar: 10 μm, *n* = 5). **E** WB for PSD95, SNAP25, SYN1, APP, and sAPPβ expression in the hippocampus (*n* = 5). **F** IHC for PSD95, SNAP25, SYN1 in the hippocampal CA3, and Aβ deposition in the hippocampus (bar: 50 μm, *n* = 5). Sham+*Ctrl*-sgRNA, female sham APP/PS1 mice treated with *Ctrl*-sgRNA; OVX+*Ctrl*-sgRNA, female ovariectomized APP/PS1 mice treated with *Ctrl*-sgRNA; OVX+*Ctrl*-sgRNA+E2, female ovariectomized APP/PS1 mice treated with *Ctrl*-sgRNA and estradiol; OVX+*Sorl1*-sgRNA+E2, female ovariectomized APP/PS1 mice treated with *Sorl1*-sgRNA and estradiol. *P < 0.05, **P < 0.01, ***P < 0.001, ns: No significance

We used CRISPR-Cas9 technology to knock down SORLA expression in the hippocampus to examine its role in how E2 mitigates the accelerated progression of AD in female APP/PS1-OVX mice. WB results verified the knockdown efficiency of SORLA (Fig. S2A). The MWM, NOR, and Y-maze tests were conducted to assess cognitive function (Fig. S2B). Neuronal SORLA knockdown inhibited the beneficial effects of E2 on cognitive function in APP/PS1-OVX mice (Fig. 3A–C; Fig. S2C). The absence of SORLA prevented the E2-induced restoration in hippocampal dendritic spine density in APP/PS1-OVX mice (Fig. 3D). WB (Fig. 3E) and IHC (Fig. 3F; Fig. S2D) results also showed that SORLA knockdown diminished E2-induced recovery in PSD95, SNAP25, and SYN1 levels. SORLA deficiency reversed the E2-induced reduction in APP and sAPPβ expression (Fig. 3E) and Aβ accumulation (Fig. 3F). Overall, these findings suggest that SORLA plays a primary role in mediating the E2-ameliorated AD progression in APP/PS1-OVX mice.

### Estradiol facilitates APP endosomal trafficking through SORLA

**Fig. 4:**
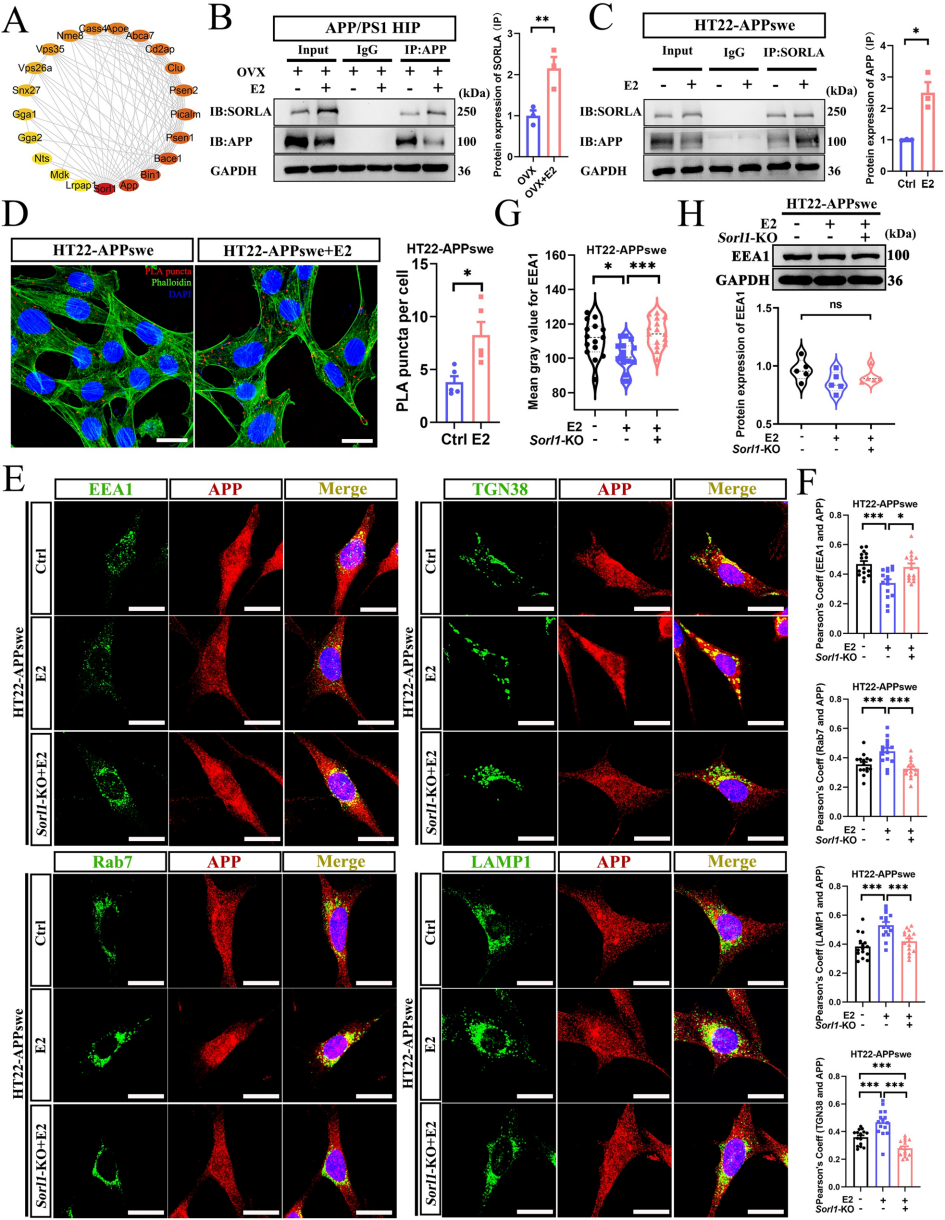
Estradiol facilitates APP endosomal trafficking through SORLA. **A** PPI network diagrams available through the STRING database and visualized through Cytoscape, where the circle layout is determined by the degree value. **B** Co-IP for APP and SORLA interactions in the hippocampus. **C** Co-IP for SORLA and APP interactions in HT22-APPswe cells. **D** PLA for APP and SORLA interactions in HT22-APPswe cells (bar: 10 μm, *n* = 5). **E**, **F** Co-localization for APP with EEA1, TGN38, Rab7, or LAMP1 in HT22-APPswe cells (bar: 20 μm, *n* = 5). **G** Quantification of the mean gray value for EEA1. **H** WB for EEA1 expression in HT22-APPswe cells (*n* = 5). OVX, female ovariectomized APP/PS1 mice; OVX+E2, female APP/PS1 mice with estradiol supplementation; Ctrl, HT22-APPswe cells treated with vehicle; E2, HT22-APPswe cells treated with estradiol; *Sorl1*-KO + E2, HT22-APPswe cells treated with estradiol after *Sorl1* knockout. *P < 0.05, **P < 0.01, ***P < 0.001

We created a protein-protein interaction (PPI) network using the STRING database and Cytoscape software to visually illustrate the interactions between *Sorl1* and its potential targets (Fig. 4A). The results indicated that SORLA may directly interact with APP, prompting us to further investigate their interaction in the presence or absence of E2. Co-immunoprecipitation (Co-IP) assays demonstrated that E2 enhanced the interaction between SORLA and APP in the hippocampus of APP/PS1-OVX mice (Fig. 4B). To further validate the interaction between SORLA and APP induced by E2 in vitro, we constructed an HT22 cell line overexpressing the APP swedish mutation (HT22-APPswe) via lentiviral transfection (Fig. S3A, B). Co-IP (Fig. 4C) and proximity ligation assay (PLA, Fig. 4D) results showed that E2 enhanced SORLA–APP interaction in HT22-APPswe cells.

SORLA interacts with various protein sorting complexes as an endosomal receptor, facilitating the transport of cargo through retromer-dependent recycling to the cell surface and endolysosomal network [30, 31]. Impaired SORLA activity leads to enlarged endosomes, which are the defining cytopathology of AD [30]. To investigate this, we generated *Sorl1*-KO cell lines from HT22-APPswe cells and the 1E3 clone was identified as homozygous (Fig. S3C, D). Immunocytochemistry (ICC) results showed that E2 decreased the area of early endosome antigen 1 (EEA1, an early endosome marker)-positive structures; however, *Sorl1*-KO inhibited this effect (Fig. 4E, F). Nevertheless, WB results showed no change in EEA1 protein levels among these groups (Fig. 4G), indicating that the increased fluorescence intensity is due to the enlarged early endosomes. The colocalization of APP with different compartments of endosomal trafficking was analyzed using an immunofluorescence assay. We observed increased colocalization of APP with EEA1, whereas colocalization with trans-golgi network protein 38 (TGN38, a TGN marker) decreased in E2-treated *Sorl1*-KO cells (Fig. 4E, H). In addition, the colocalization of APP with ras-related protein 7 (Rab7, a maturing endosome marker) and Lysosomal-associated membrane protein 1 (LAMP1, a lysosome marker) also decreased in E2-treated *Sorl1*-KO cells (Fig. 4E, H). These data indicate that E2 facilitates the non-amyloidogenic pathways of APP in both the endosomal-TGN and endosomal-lysosomal trafficking via SORLA, ultimately reducing Aβ generation.

### Estradiol increases SORLA expression through ERα in HT22-APPswe cells

**Fig. 5:**
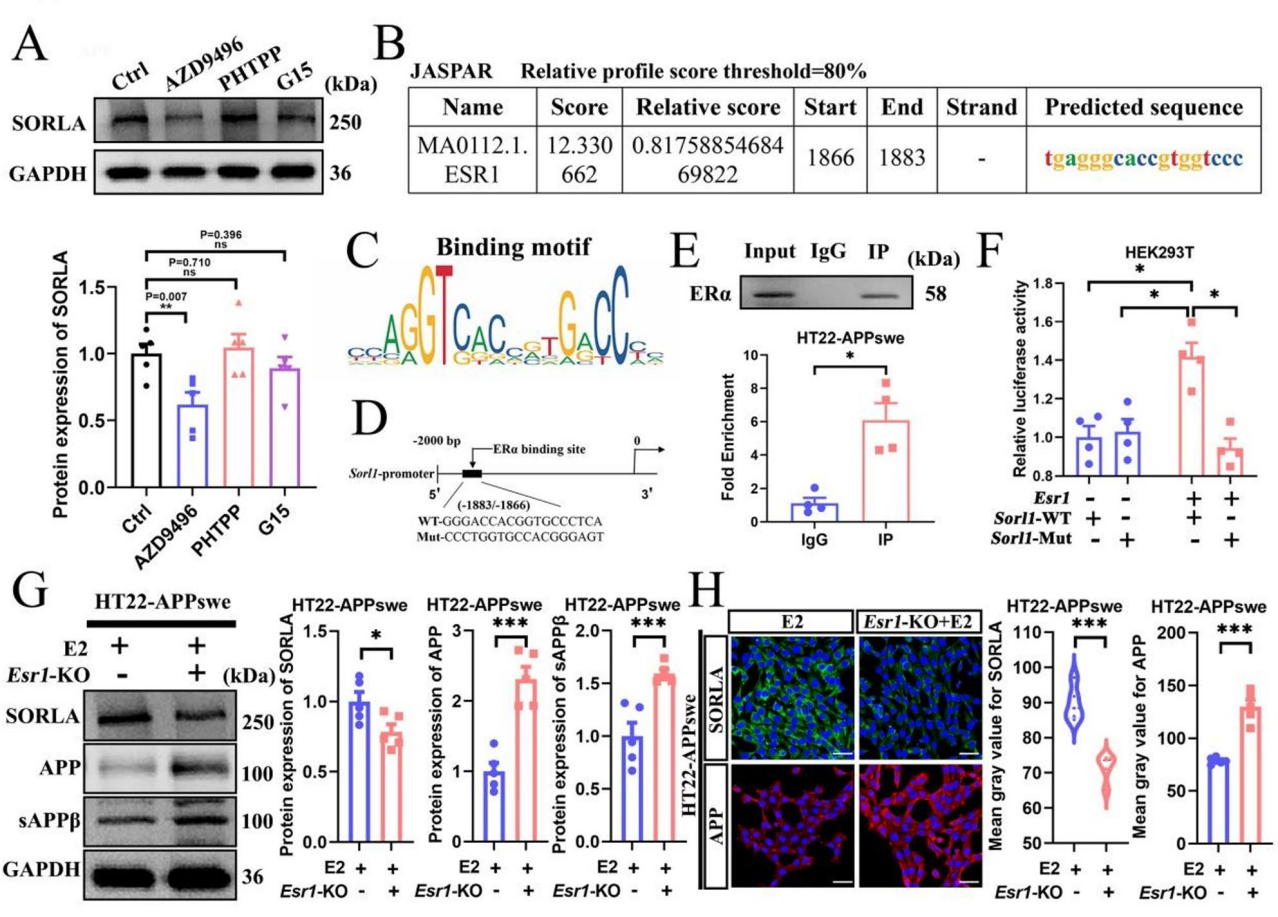
Estradiol increases SORLA expression through ERα in HT22-APPswe cells. **A** WB for SORLA expression in HT22-APPswe cells treated with vehicle (Ctrl), AZD9496 (ERα inhibitor), PHTPP (ERβ inhibitor), or G15 (GPER1 inhibitor). **B** Using the UCSC and JASPAR databases to predict the binding sites between ERα and the *Sorl1* promoter. **C** The binding motif sequence of ERα. **D** The ERα binding site of *Sorl1* promoter. **E** ChIP-qPCR for the ERα binding site of *Sorl1* promoter in HT22-APPswe cells. **F** Dual-luciferase assay for WT or Mut *Sorl1* promoter activity in HEK-293T cells transfected with *Esr1* plasmid or control vector. **G** WB for SORLA, APP, and sAPPβ expression in HT22-APPswe cells with or without *Esr1*-KO (*n* = 5). **H** ICC for SORLA and APP expression in HT22-APPswe cells with or without *Esr1*-KO (bar: 50 μm, *n* = 5). *Esr1*-KO, HT22-APPswe cells treated with estradiol after *Esr1* knockout. *P < 0.05, ***P < 0.001

To clarify the mechanism that E2 increased SORLA expression, we treated HT22-APPswe cells with AZD9496 (an ERα inhibitor), PHTPP (an ERβ inhibitor), or G15 (a GPER1 inhibitor). WB results indicated that AZD9496 effectively blocked E2’s ability to promote SORLA expression, but not in the PHTPP and G15 groups (Fig. 5A). Additionally, IHC and WB analysis demonstrated that E2 administration significantly attenuated the decrease in ERα expression in both the hippocampus and cerebral cortex following ovariectomy (Fig. S4A–C). These results suggested that E2 may enhance SORLA expression via ERα. To further determine whether the SORLA upregulation is associated with the transcriptional activation of ERα, we used the UCSC and JASPAR databases to identify potential binding sites between mouse ERα and the *Sorl1* promoter. We identified one potential binding site and the binding intensity score, start and end points of the binding segment, and associated gene sequences are illustrated in Fig. 5B − D. The chromatin immunoprecipitation (ChIP)-qPCR results demonstrated that ERα interacted with the *Sorl1* promoter in the − 1883 to − 1866 bp region (Fig. 5E). Moreover, dual-luciferase reporter gene assay results indicated that E2 increased *Sorl1* promoter activity via ERα in HEK293T cells (Fig. 5F), highlighting the importance of ERα targeting the specific *Sorl1* promoter region for mediating the effects of E2. To further clarify the role of ERα in E2 regulation of SORLA expression, we constructed *Esr1*-KO HT22-APPswe cells and identified the 1C5 line as homozygous (Fig. S3E, F). WB and ICC results indicated that *Esr1*-KO reversed the upregulation of SORLA, as well as the downregulation of APP and sAPPβ in E2-treated HT22-APPswe cells (Fig. 5G, H). Notably, ERα overexpression significantly increased SORLA protein levels upon E2 treatment in *Esr1*-KO HT22-APPswe cells, as shown in Fig. S4D and E. These data indicate that E2 increases SORLA expression and reduces Aβ pathology through ERα.

### The effect of estradiol mediated by ERα on ameliorating AD progression is dependent on SORLA in female APP/PS1-OVX mice

**Fig. 6:**
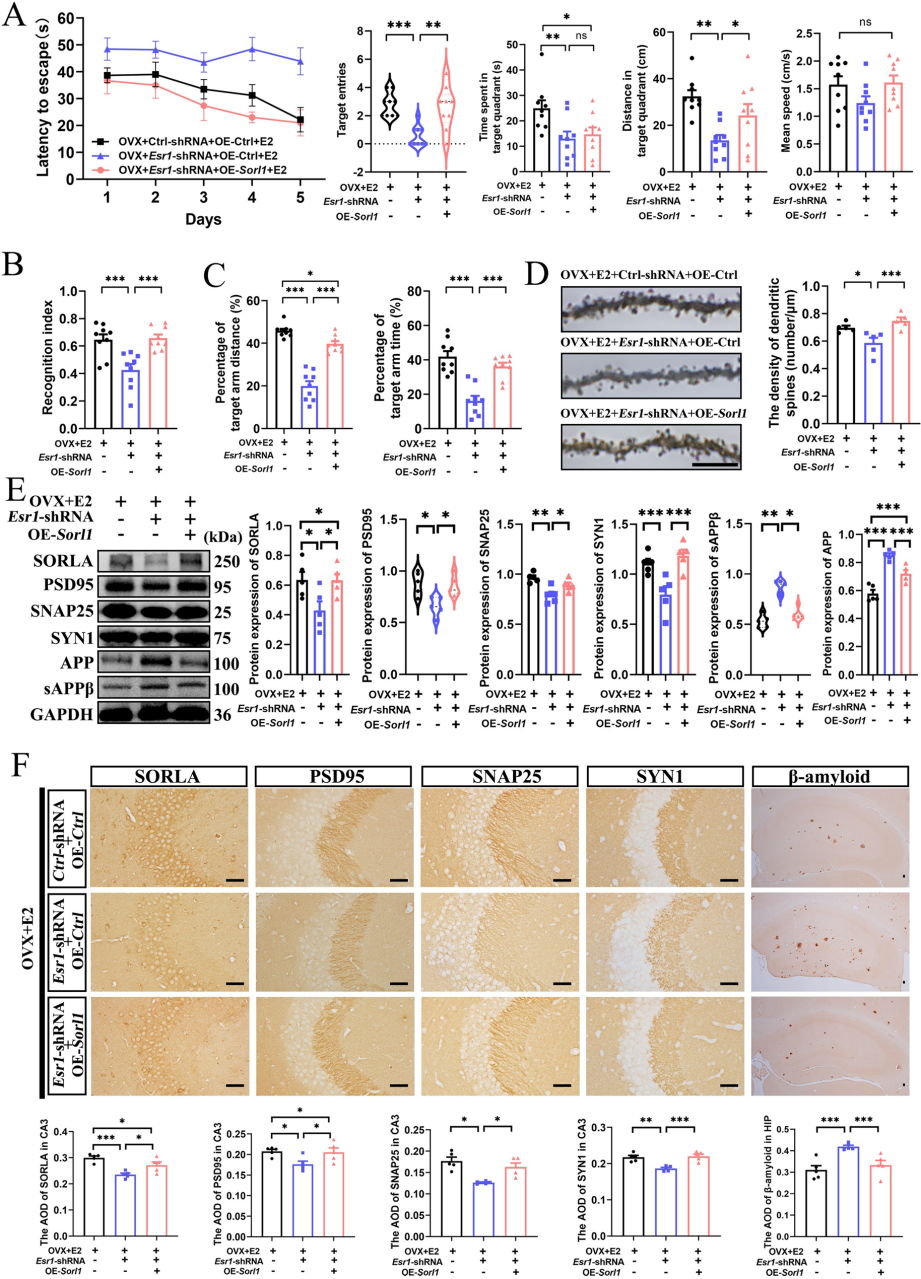
The effect of estradiol mediated by ERα on ameliorating the AD progression is dependent on SORLA in female APP/PS1-OVX mice. **A** The latency to find the hidden platform during training trials, as well as the target entries, the time spent and distance in the target quadrant, and the mean speed during probe trials in the MWM (*n* = 9). **B** Recognition index in the NOR (*n* = 9). **C** The distance and time spent in target (novel) arm in the Y maze (*n* = 9). **D** Golgi staining for the hippocampal dendritic spine density (bar: 10 μm, *n* = 5). **E** WB for SORLA, PSD95, SNAP25, SYN1, APP, and sAPPβ expression in the hippocampus (*n* = 5). **F** IHC for SORLA, PSD95, SNAP25, SYN1 in the hippocampal CA3, and Aβ deposition in the hippocampus (bar: 50 μm, *n* = 5). OVX+*Ctrl*-shRNA + OE-*Ctrl*+E2, female ovariectomized APP/PS1 mice treated with *Ctrl*-shRNA, OE-*Ctrl*, and E2; OVX+*Esr1*-shRNA + OE-*Ctrl*+E2, female ovariectomized APP/PS1 mice treated with *Esr1*-shRNA, OE-*Ctrl*, and E2; OVX+*Esr1*-shRNA + OE-*Sorl1* + E2, female ovariectomized APP/PS1 mice treated with *Esr1*-shRNA, OE-*Sorl1*, and E2. *P < 0.05, **P < 0.01, ***P < 0.001, ns: No significance

To clarify the role of SORLA in estradiol’s ERα-mediated improvement of AD progression, we over-expressed SORLA in the hippocampus of female APP/PS1-OVX mice with ERα knockdown (Fig. S5A). The efficiency were confirmed by WB (Fig. S5B, C). Behavioral studies results showed that ERα knockdown increased latency to the escape platform during training and decreased target entries during the test phase in the MWM test, decreased recognition index in the NOR test, and shorter distances traveled and less time spent in the target arm during the test phase in the Y maze test; however, SORLA over-expression reversed these effects (Fig. 6A–C; Fig. S5D). Overexpression of SORLA restored the reduced levels of hippocampal dendritic spine density, SORLA, PSD95, SNAP25, and SYN1, and also reversed the elevated levels of Aβ, APP, and sAPPβ in the hippocampus of *Esr1* shRNA-treated female APP/PS1-OVX mice (Fig. 6D − F; Fig. S5E). These findings highlight the importance of SORLA in the estradiol’s ERα-mediated improvement of AD progression.

## Discussion

This study enhances our understanding of the protective effects of E2 against AD and highlights its contribution to the sex differences observed in the disease. E2 alleviates the accelerated progression of AD caused by OVX in female APP/PS1 mice. Mechanistically, E2 prevents the decline in SORLA levels following ovariectomy via ERα, promoting the non-amyloidogenic pathways of APP in both the endosomal-TGN and endosomal-lysosomal trafficking, which reducing Aβ accumulation.

Several studies have shown that postmenopausal women receiving hormonal replacement therapy have slower cognitive decline and a lower risk of AD [32, 33]; however, not all studies have shown these benefits. In a cross-sectional study, elder female users (aged > 70 years) of hormonal replacement therapy were found to have faster tau accumulation in the entorhinal cortex, inferior temporal, and fusiform gyri compared to non-users; however, the association between hormonal replacement therapy and tauopathy in younger women was negligible [34]. There continues to be debate about the timing of E2 supplementation for AD during menopause, which is referred to as the “window of opportunity”. The concept of the “window of opportunity” for estrogen therapy in AD has emerged from clinical and preclinical studies suggesting that E2 exerts neuroprotective effects only when administered during a critical period near menopause, typically within 5–10 years of ovarian hormone loss [35]. Beyond this window, late-life hormone replacement therapy has shown limited or even adverse effects on cognitive outcomes, likely due to age-related changes in estrogen receptor expression, mitochondrial function, and neuroinflammatory status [36]. This temporal constraint, combined with increased risks of breast cancer, endometrial hyperplasia, and thromboembolic events, significantly limits the clinical utility of E2-based interventions, particularly for male patients and postmenopausal women outside the optimal treatment window. Consequently, there is an urgent need for therapeutic strategies that can bypass estrogen signaling entirely while preserving or enhancing its beneficial downstream effects—such as the regulation of SORLA expression and function.

In vivo studies shown that OVX causes E2 deficiency, which worsens learning and memory in APP/PS1 mice [37]. Additionally, E2 reduced Aβ accumulation in 3xTg-AD OVX mice [26, 38]. Our findings align with those of previous research, showing that E2 improves cognitive deficits, synaptic dysfunction, and Aβ deposition in female APP/PS1-OVX mice. While the OVX mouse model provides a controlled and reproducible system for studying the effects of acute estrogen loss, it is important to acknowledge its limitations in recapitulating the full complexity of natural menopause [27]. Unlike surgical menopause, natural menopause involves a gradual decline in ovarian hormones over years, accompanied by systemic physiological adaptations, including changes in neuroimmune signaling and metabolic regulation [39]. These differences suggest that our findings from the OVX model may not fully capture the nuanced hormonal and physiological dynamics of natural menopause. Future studies utilizing aging models, perimenopausal transition models, or hormone gradient simulations will be valuable to validate and extend our observations under conditions that more closely mimic the natural menopausal process.

Despite significant efforts to understand amyloid and other pathological events in AD, current interventions have not been effective in slowing disease progression [15, 40]. Recent genetic studies on late-onset AD have identified numerous risk genes related to endosomal trafficking [41]. Enlarged endosomes and dysfunctional endosomal networks were believed to be very early cytopathological signs in AD neurons, occurring even in the absence of Aβ deposition and neurofibrillary tangles [42, 43]. Additionally, dysfunction of endosomal networks promotes Aβ accumulation and the development of tauopathies [44–46]. Our results indicate that E2 reduces the endosomal volume and facilitates the transport of APP from early endosome to the Golgi apparatus and lysosomes, leading to reduced APP level and Aβ deposition in HT22-APPswe cells. This finding underscores E2’s critical role in reducing endosomal dysfunction in AD pathology, offering new directions for the development of targeted therapies.

The SORLA protein, encoded by *Sorl1*, is a sorting receptor involved in endocytic sorting and trafficking 47]. Missense mutations in *Sorl1* have been identified in patients with AD, and these mutations contribute to the dysfunction of endosomal networks, which is directly associated with the pathophysiology of the disease [30, 48, 49]. Our study showed that E2 increased SORLA protein expression in the hippocampus and cerebral cortex, enhancing the SORLA–APP interaction. SORLA interacted with APP to promote its non-amyloidogenic pathway, thereby reducing Aβ production. The absence of SORLA diminished the positive effects of E2 on cognitive dysfunction, synaptic damage, and Aβ deposition in APP/PS1-OVX mice. Furthermore, it disrupted the transport of APP from endosomes to the Golgi apparatus and lysosomes, resulting in enlarged endosomes and heightened Aβ production in HT22-APPswe cells. The above results indicate that E2 promotes the endosomal transport process of APP by upregulating SORLA, thereby reducing Aβ accumulation.

In addition to the molecular mechanisms elucidated in this study, our findings support the emerging concept that elevating SORLA expression may represent a viable therapeutic strategy for AD that is applicable to both men and women. While E2 has been shown to modulate SORLA levels in this study, its clinical utility is limited by sex-specific side effects and risks associated with long-term hormone therapy. Therefore, identifying alternative strategies—such as transcriptional activation via FOXP1 [50], pharmacological enhancement of retromer function [51], or inhibition of the PKCι/λ-βARR2 axis [52]—offers the potential to bypass E2-related limitations. These approaches could provide broader and safer therapeutic options for both sexes by directly targeting SORLA expression and trafficking pathways that are critical for endosomal recycling and amyloid clearance. Future studies should aim to develop and test SORLA-elevating interventions in both male and female preclinical models to ensure efficacy and safety across diverse populations.

Recent findings suggest that impairment of estrogen receptor signaling is related to the AD pathogenesis [53]. ERα and ERβ uniquely influence tau phosphorylation in AD through the miR-218/PTPA pathway [54]. In female 5xFAD mice, activating the estrogen receptor GPER1 significantly enhances recognition memory after 24 h; however, male mice showed no such effect [55]. In this study, we demonstrated that the ERα inhibitor AZD9496 significantly inhibited E2-induced SORLA expression. We predicted an ERα binding site in the *Sorl1* promoter region using the UCSC and JASPAR databases. Subsequently, we designed primers and mutation plasmids targeting this site. Using ChIP-qPCR and luciferase reporter assays, we confirmed that this site mediates the regulation of SORLA expression by E2 through ERα. While the HT22 cell line enabled detailed mechanistic dissection of the ERα-SORLA pathway, future studies utilizing primary hippocampal neuron cultures would be valuable to further validate these findings under more heterogeneous conditions. In addition, the effect of E2 mediated by ERα on ameliorating AD progression is dependent on SORLA in female APP/PS1-OVX mice. The in vivo rescue experiments—demonstrating that SORLA overexpression ameliorates cognitive deficits, synaptic dysfunction, and Aβ pathology in ERα-knockdown AD mice—provide causal evidence that SORLA is a key downstream effector of ERα and a critical mediator of AD-related phenotypes. These findings complement our in vitro mechanistic dissection and establish a functional link between the ERα-SORLA axis and AD pathology in the physiologically relevant context of the mammalian brain. While our study confirms ERα binding to the *Sorl1* promoter, the specific coactivators (e.g., p160 family [56], p300/CBP [57]) and regulatory elements (e.g., AF-1-Dependent Elements [58]) potentially involved remain to be characterized, representing a limitation that warrants future investigation.

Our study reveals that the therapeutic potential of E2 for ameliorating AD progression both in vivo and in vitro. By enhancing SORLA expression through ERα, E2 promotes APP endosomal trafficking, which reduces Aβ production and ameliorates synaptic dysfunction and cognitive deficits in AD. These findings elucidate the role of E2 in regulating endosomal trafficking in AD pathology and shed light on the potential mechanisms contributing to sex differences in the disease.

## Materials and methods

### Animals and treatment

We obtained 4-month-old female APP/PS1 mice from HFK BIOSCIENCE CO., Ltd. (Beijing, China), which carry the human transgene with the APP Swedish mutation (K595N/M596L) and PS1 deltaE9. Mice were housed at 22–25 °C with 55 ± 5% humidity. They were kept on a 12-h light-dark cycle and had access to a standard diet and water. All animal procedures were conducted in accordance with the ARRIVE guidelines and received formal approval from the Institutional Ethics Committee at Hebei Medical University (Approval Permit No. IACUC-Hebmu-2023049). All methods were carried out in accordance with relevant guidelines and regulations.

Mice were anesthetized using 2% isoflurane and randomly assigned to either the sham operation or bilateral OVX group. The OVX procedure involved a midline dorsal incision, followed by the removal of ovaries under aseptic conditions. Seven days after the OVX procedure, some mice received an injection of E2 (10 µg/kg/d, i.p., HY-B0141R, MCE, USA).

Mice were stereotactically injected with three types of viruses into the hippocampal region (AP = − 2.0 mm, ML = ± 1.5 mm, DV = − 1.6 mm): AAV9-hSyn-Sorl1-sgRNA (TCACCGCGTGGAAGGCTTACA) and its control virus for SORLA knock down, AAV9-hSyn-*Esr1*-shRNA (GCAGTAACGAGAAAGGAAACA) and its control virus for ERα knock down, as well as AAV9-hSyn-*Sorl1*-sgRNA (GCCGTGCTCATTTCCCCGGG) and its control virus for SORLA over-expression from GENECHEM (Shanghai, China). A 1 µl viral solution with a concentration of 1 × 10^8^ TU/ml was injected at each site at a rate of 0.1 µl/min, and the needle was held in place for 5 min before withdrawal. Seven days after stereotactic injection, the mice underwent OVX.

### Morris water maze

A circular pool (120 cm in diameter) was divided into four equal quadrants filled with water at a temperature of 22 ± 2 °C, with a platform (10 cm in diameter) submerged 1 cm below the water surface to serve as the target for the mice. During the spatial acquisition phase, mice underwent training for 5 days, completing four trails each day, with each trial lasting 60 s. If the mice were unable to locate the platform within 60 s, they were placed on the platform for a duration of 10 s. In the probe trial, after removing the platform, the mice were positioned on the side of the pool opposite the target quadrant and allowed to swim freely for 60 s. We recorded and analyzed the total distance and time spent in the target quadrant, the number of target entries, and the mean speed.

### Novel object recognition

The NOR assessment was performed in a white cube opaque box 45 cm × 45 cm × 45 cm. In the habituation phase, mice were allowed to explore the box freely for 10 min. The box contained two identical objects located in the upper corners. In the testing session, one of the objects was replaced with a different object in its original location. The mice were then returned to the box and allowed to explore freely for 10 min. The recognition index was calculated by dividing the time spent exploring a different object by the total exploration time.

### Y maze

The Y maze consisted of three arms, measuring 8 × 30 × 15 cm each, designated as the start, target (novel), and third arm. The experiment included training and testing phases. During the training phase, mice were allowed to explore for 10 min only in the start and third arms, while the target arm was blocked. The testing phase occurred 4 h after the training. In the test phase, mice were returned to the start arm and given 10 min of free access to all three arms. The total distance and time spent in the target arm were recorded and analyzed.

### Immunohistochemistry

Mice were anesthetized using 2% isoflurane and then underwent cardiac perfusion with phosphate buffered saline (PBS), followed by 4% paraformaldehyde. Brain tissue was collected and sectioned into 10-µm thick slices. The sections were stained with the mouse/rabbit IHC kit (Proteintech, PK10006, Wuhan, China). After dewaxing and hydration, the sections were washed three times with PBS and then blocked with 5% goat serum for 30 min. The sections were incubated with primary antibodies against PSD95, SNAP25, SYN1, SORLA, and Aβ (listed in Table S2) at 4 °C overnight. HRP-conjugated goat anti-mouse or anti-rabbit antibody was incubated at room temperature for 1 h. After thorough washing with PBS, chromogenic detection was achieved using DAB. The images were acquired using an Olympus BX53 Imaging System. The average optical density values (AOD) were determined using ImageJ software.

### Western blotting

Hippocampus tissue samples and cells were processed using RIPA buffer (R0010, Solarbio, Beijing, China) supplemented with the protease inhibitor PMSF (IKM1140, Solarbio, China). The samples were then centrifuged at 12,000 rpm for 20 min at 4 °C. The supernatant was diluted with loading buffer and then boiled at 95 °C for 5 min. After boiling, the mixture stored at − 80 °C. Proteins were separated using SDS-PAGE gels and subsequently transferred to PVDF membranes (03010040001, Roche, Switzerland). After blocking with 5% nonfat milk for 2 h, the membranes were incubated at 4 °C overnight with primary antibodies against PSD95, SNAP25, SYN1, SORLA, ERα, APP, sAPPβ, and GAPDH (listed in Table S2). Then, they were incubated at room temperature with secondary HRP-conjugated goat anti-mouse/rabbit antibodies. Bands were visualized using a Bio-Rad ChemiDocTM Touch Imaging System. We used ImageJ to measure the grayscale values of the bands.

### Golgi staining

Brain tissues were collected for the Golgi staining test according to the user manual of the Golgi staining kit (GMS80020.1 v.A, GENMED SCIENTIFICS INC., USA). The brains were immediately immersed in mixture of reagents A and B and stored in the dark at room temperature for 2 weeks before incubating with reagent H at 4 °C for 1 week. After sectioning the brains into 100 μm coronal slices, they were stained with mixed reagent I/J. The stained pyramidal neurons in the hippocampal region were imaged using the Olympus BX53 Imaging System. Dendritic spine density was analyzed using Fiji software.

### Cell culture and treatment

We obtained mouse hippocampal neuronal line HT22 and human embryonic kidney HEK293T cells from Cyagen Biosciences Inc. (Suzhou, China). We cultured the cells in Dulbecco’s Modified Eagle’s Medium (DMEM) supplemented with 10% fetal bovine serum (FBS) and 5% Penicillin-Streptomycin. The culture was maintained at 37 °C in a humidified atmosphere with 5% CO2. The cells were treated with E2 (10 nM) or DMSO for 24 h for RNA detection and 48 h for protein detection.

The *Sorl1* knockout (KO) and *Esr1* KO HT22 cell lines were generated by Cyagen Biosciences Inc. using CRISPR/Cas9 gene editing technology mediated by electroporation. We utilized two sgRNAs targeting *Sorl1*: gRNA-B1(GCTGTACTGTGCCTGAGAGC-AGG), gRNA-A2 (TAAGTCCTGACCTGGTGTAC-TGG), and two sgRNAs targeting *Esr1*: gRNA-A1 (TTCTTCAAGGTTAATATAAC-AGG), gRNA-B2 (CAGGAGAAGTGGTAATGCCA-GGG).

The HT22-APPswe cell line was established using lentiviral infection. The APP K595N/M596L fragment, which contains the human APPswe Mutation, was inserted into a pHBLV-CMV-MCS-3flag-EF1-puro vector (HanBio Biotechnology, Shanghai, China). HT22 cells were transfected and then screened with puromycin.

### Immunocytochemistry

The cells on the coverslips were washed three times with PBS and then fixed in 4% paraformaldehyde for 10 min. Next, the cells were permeabilized with 0.1% Triton X-100 for 10 min, followed by blocking with 10% donkey serum albumin in PBS for 1 h at room temperature. The cells were incubated overnight at 4 °C with primary antibodies targeting SORLA, APP, PSD95, SNAP25, SYN1, TGN38, EEA1, Rab7, and LAMP1 (listed in Table S2). Following three 10-min washes with PBS, the cells were incubated in the dark with Alexa Fluor 488-labeled donkey anti-rabbit or Alexa Fluor 647-labeled donkey anti-mouse antibody for 1 h at room temperature. Finally, the coverslips were mounted using DAPI Fluoromount-G (0100 − 20, SouthernBiotech, USA). Fluorescence signals were captured using an Olympus FV1200 confocal microscope. ImageJ was used to measure the mean gray value.

### RNA sequencing

Total RNA was extracted from the mouse hippocampus using TRIzol^®^ Reagent (15596026CN, Invitrogen™, USA) following manufacturer’s instructions. The library preparations were performed using an Illumina Novaseq 6000 scanner. Differential expression analysis was performed using DESeq2 (v1.20.0), considering genes with |log2 Fold Change| > 0.58 and P-value < 0.05 as DEGs. We used the clusterProfiler R software package for the GO function enrichment analysis. A GO function was deemed significantly enriched when *P*-value < 0.05.

### Quantitative real-time PCR

Total RNA was extracted from mouse hippocampus or HT22-APPswe cells using TRIzol^®^ Reagent following manufacturer’s instructions. For reverse transcription analysis, we used HiScript III RT SuperMix for qPCR (gDNA wiper) from Vazyme (R323-01, Nanjing, China). Quantitative RT-PCR was performed using the ChamQ Uiversal SYBR qPCR Master Mix (Q711, Vazyme). The primer sequences used in this study are listed in Table S3.

### Construction of protein-protein interaction networks and screening targets

*Sorl1* was entered into the STRING database (https://string-db.org), where the analysis focused on “Mus musculus,” with a confidence score of 0.4 and a maximum of 20 interactors, along with default parameters. The network from the STRING database was then brought into Cytoscape software (https://cytoscape.org/, version 3.10.0) to calculate each node’s parameters in the network diagram and show the molecular connections.

### Co-immunoprecipitation

Whole cell or mouse hippocampus lysates were collected following the manufacturer’s instructions (P2179M, Beyotime, Beijing, China) and then centrifuged at 12,000 rpm for 20 min at 4 °C. The lysates were incubated overnight at 4 °C with APP, SORLA, or IgG antibody (listed in Table S2). After additional incubation with Protein A + G Magnetic Beads for 2 h at room temperature, the beads were washed with IP lysis solution, then eluted and heated for 5 min at 95 °C with SDS sample loading buffer for WB.

### Proximity ligation assay

Cells cultured on coverslips were washed three times with PBS and then fixed in 4% paraformaldehyde for 10 min. The Duolink^®^ In Situ (Red) Kit (DUO92101, Sigma-Aldrich, USA) was used for the PLA. Cells were permeabilized with 0.1% Triton X-100 for 10 min and blocked at 37 °C for 30 min. Cells were then incubated with APP and SORLA antibodies (listed in Table S2) overnight at 4 °C. The following day, cells were washed twice for 5 min each time with Wash buffer A. They were then incubated with Duolink In Situ PLA anti-rabbit PLUS and anti-mouse MINUS PLA Probe at 37 °C for 1 h, followed by another wash with Wash buffer A. After incubation in ligation solution at 37 °C for 30 min, cells were washed with Wash buffer (A) Then, they were incubated in amplification solution at 37 °C for 100 min, followed by two washes of 10 min each in Wash buffer (B) The cytoskeleton was stained with phalloidin-fluor 488 reagent (abs47048271-300T, Absin, Shanghai, China). Finally, the coverslips were mounted with DAPI Fluoromount-G, and fluorescence signals were captured using a confocal microscope. The PLA puncta per cell were analyzed using ImageJ software.

### Chromatin immunoprecipitation-qPCR

Chromatin was collected from cells following the manufacturer’s protocols outlined in the ChIP-IT High Sensitivity (53040, Active Motif, USA). HT22-APPswe cells were treated with E2 (10 nM) for 24 h, followed by cell fixation, chromatin sonication, immunoprecipitation, and DNA purification. The chromatin was incubated overnight at 4 °C with ERα antibody. Rabbit IgG antibody was used as a negative control (listed in Table S2). The immunoprecipitated fraction was subsequently analyzed by qRT-PCR to assess the results. The primer sequences used in this study are listed in Table S3.

### Dual-luciferase reporter assay

We introduced the synthetic *Sorl1* promoter gene (wild type, WT) and its mutated sequence (Mut) into the PEZX-FR01 vector (General Biol, Chuzhou, China). The WT and Mut luciferase reporter plasmids were co-transfected with the *Esr1* expression plasmid (mEsr1 pcDNA3.1-HA, General Biol) and a blank plasmid control (pc) into HEK293T cells, followed by treatment with E2 (10 nM). After 48 h, we collected and lysed the cells. Luciferase activity was measured using a Luciferase Detection Kit (E1910, Promega, USA). Subsequently, absorbance measurements were conducted using a microplate reader.

### Statistical analysis

All data are expressed as as mean ± SEM or median (25th percentile; 75th percentile). Statistical analysis was performed using SPSS 23.0 (IBM Corp., Armonk, NY, USA). Data following a normal distribution were compared using the *Independent Samples test* between the two groups, while those did not were assessed using *Mann–Whitney U test*. Comparisons among three or more groups of data that follow a normal distribution were conducted by a one-way ANOVA using *LSD* or *Dunnett T3*, if not were assessed using *Kruskal–Wallis H*. The MWM test statistical analysis employed two-way ANOVA followed by *Tukey’s multiple comparison test*. A significance level of *P* < 0.05 was considered statistical significance.

## Supplementary Information

Supplementary Material 1.

## Data Availability

All data generated or analysed during this study are included in this published article (and its supplementary files), further inquiries can be directed to the corresponding author upon reasonable request.
