# Supplementary material for: Estradiol ameliorates AD pathology and cognitive deficits by SORLA-mediated APP endosomal trafficking
Source: Alzheimers Res Ther. 2026 Mar 25;18:100. doi: 10.1186/s13195-026-02027-2 (PMC13137737; doi:10.1186/s13195-026-02027-2)

Supplementary figures

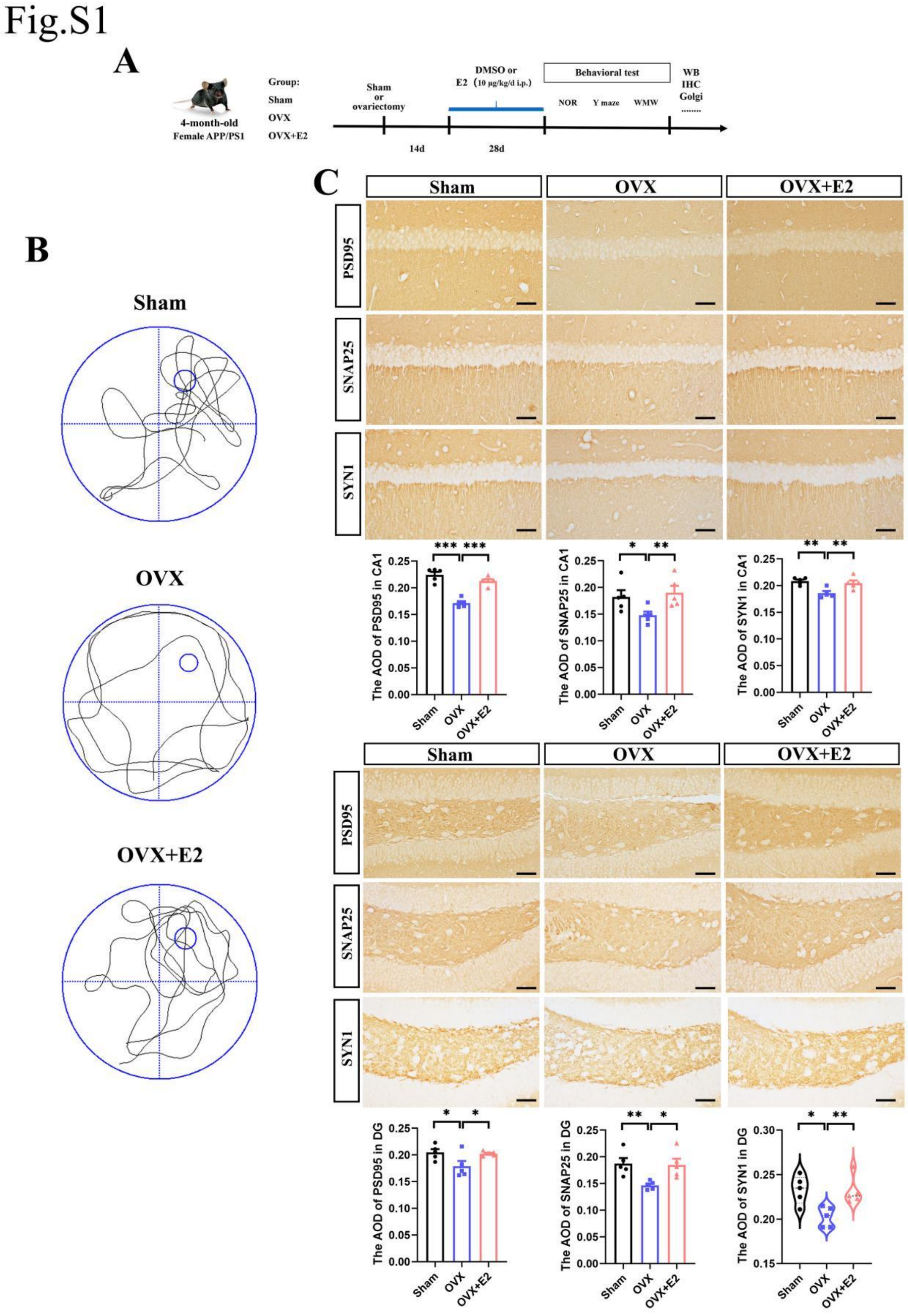

Fig. S1. Estradiol improves synaptic damage in female APP/PS1-OVX mice. (A) Experimental procedures of ovariectomy, estradiol administration and behavioral tests.

(B) Track plots of MWM. (C) IHC for PSD95, SNAP25, and SYN1 expression in the hippocampal CA1 and DG regions (bar: 50 μm, n = 5). Sham, female sham APP/PS1 mice; OVX, female ovariectomized APP/PS1 mice; OVX+E2, female ovariectomized APP/PS1 mice with estradiol supplementation. *P < 0.05, **P < 0.001, ***P < 0.001.

Fig.S2

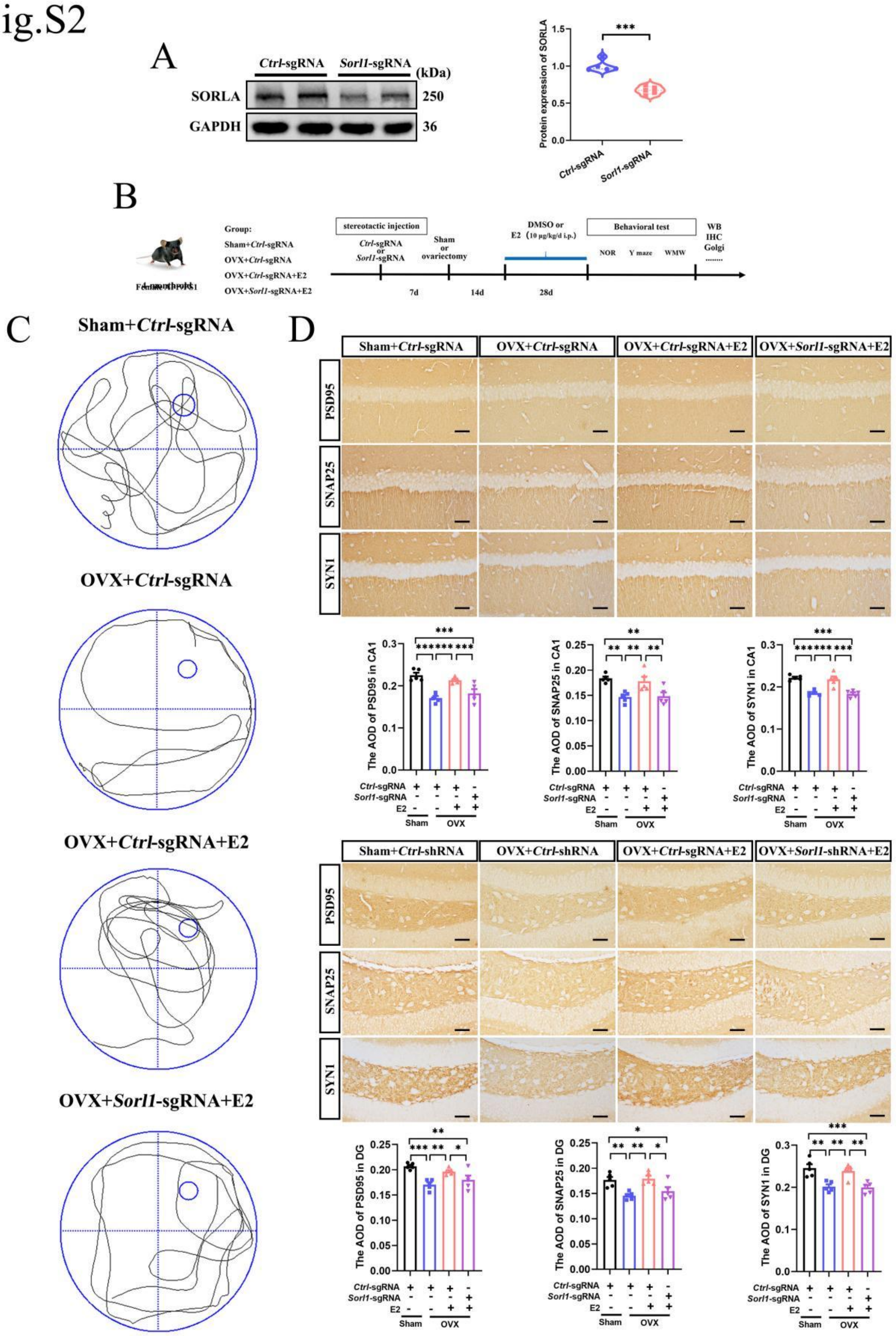

Fig. S2. Estradiol improves synaptic damage in female APP/PS1-OVX mice via SORLA. (A) WB for SORLA expression in APP/PS1-OVX mice treated with *Ctrl*-sgRNA or *Sorl1*-sgRNA. (B) Experimental procedures of ovariectomy, estradiol administration, virus injection, and behavioral tests. (C) Track plots of MWM. (D) IHC for PSD95, SNAP25, and SYN1 expression in the hippocampal CA1 and DG regions (bar: 50 μm, n = 5). Sham+*Ctrl*-sgRNA, female sham APP/PS1 mice treated with *Ctrl*-sgRNA; OVX+*Ctrl*-sgRNA, female ovariectomized APP/PS1 mice treated with *Ctrl*-sgRNA; OVX+*Ctrl*-sgRNA+E2, female ovariectomized APP/PS1 mice treated with *Ctrl*-sgRNA and estradiol; OVX+*Sorl1*-sgRNA+E2, female ovariectomized APP/PS1 mice treated with *Sorl1*-sgRNA and estradiol. **P* < 0.05, ***P* < 0.001, ****P* < 0.001.

Fig.S3

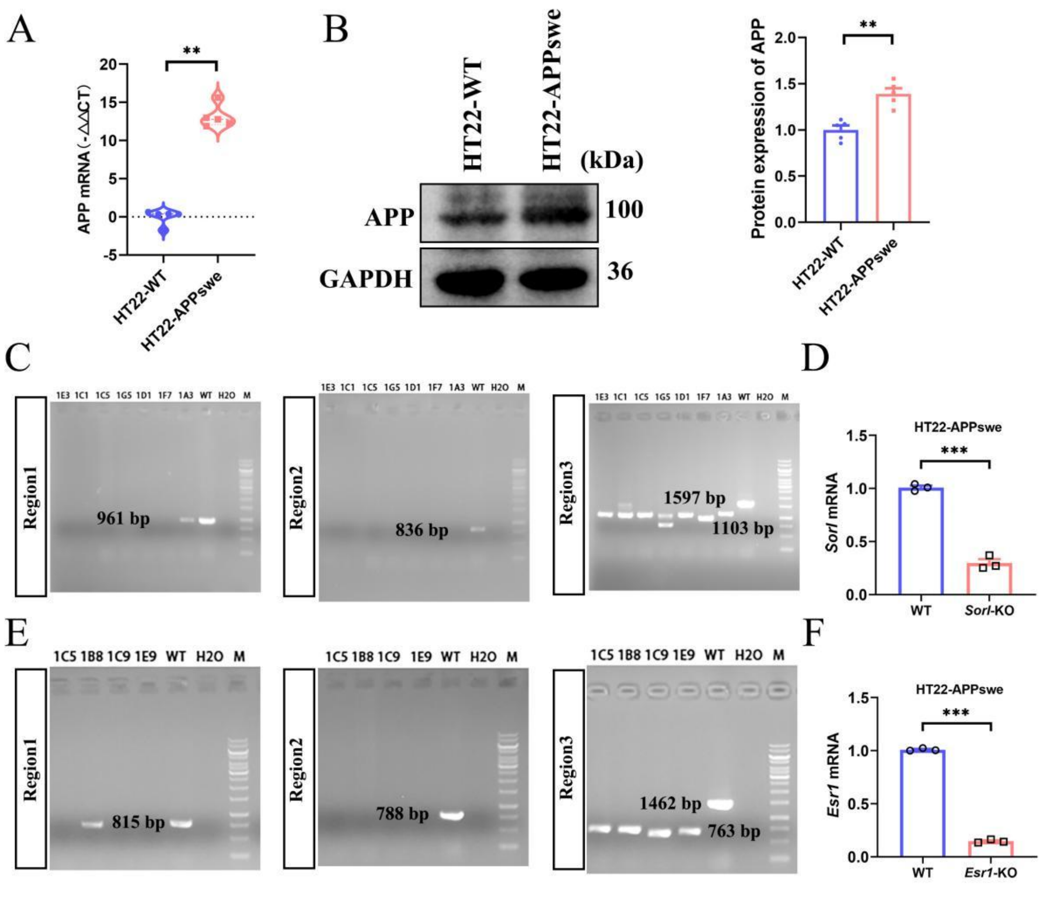

Fig. S3. Construction of HT22-APPswe cells, *Sorl1*-KO HT22-APPswe cells, and *Esr1*-KO HT22-APPswe cells. (A) Quantitative RT-PCR for APP mRNA in HT22-WT and HT22-APPswe cells. (B) WB for APP expression in HT22-WT and HT22-APPswe cells. (C) Detection of *Sorl1*-KO cell clones construction by PCR with

primer pairs: Region 1 F/R (upstream incision), Region 2 F/R (downstream incision), and Region 3 F/R (full length). (D) Quantitative RT–PCR for *Sorl1* mRNA expression in WT and *Sorl1*-KO HT22-APPswe cells. (E) Detection of *Esr1*-KO cell clones construction by PCR with primer pairs: Region 1 F/R (upstream incision), Region 2 F/R (downstream incision), and Region 3 F/R (full length). (F) Quantitative RT-PCR for *Esr1* mRNA in WT and *Esr1*-KO HT22-APPswe cells. ***P* < 0.001, ****P* < 0.001.

Fig.S4

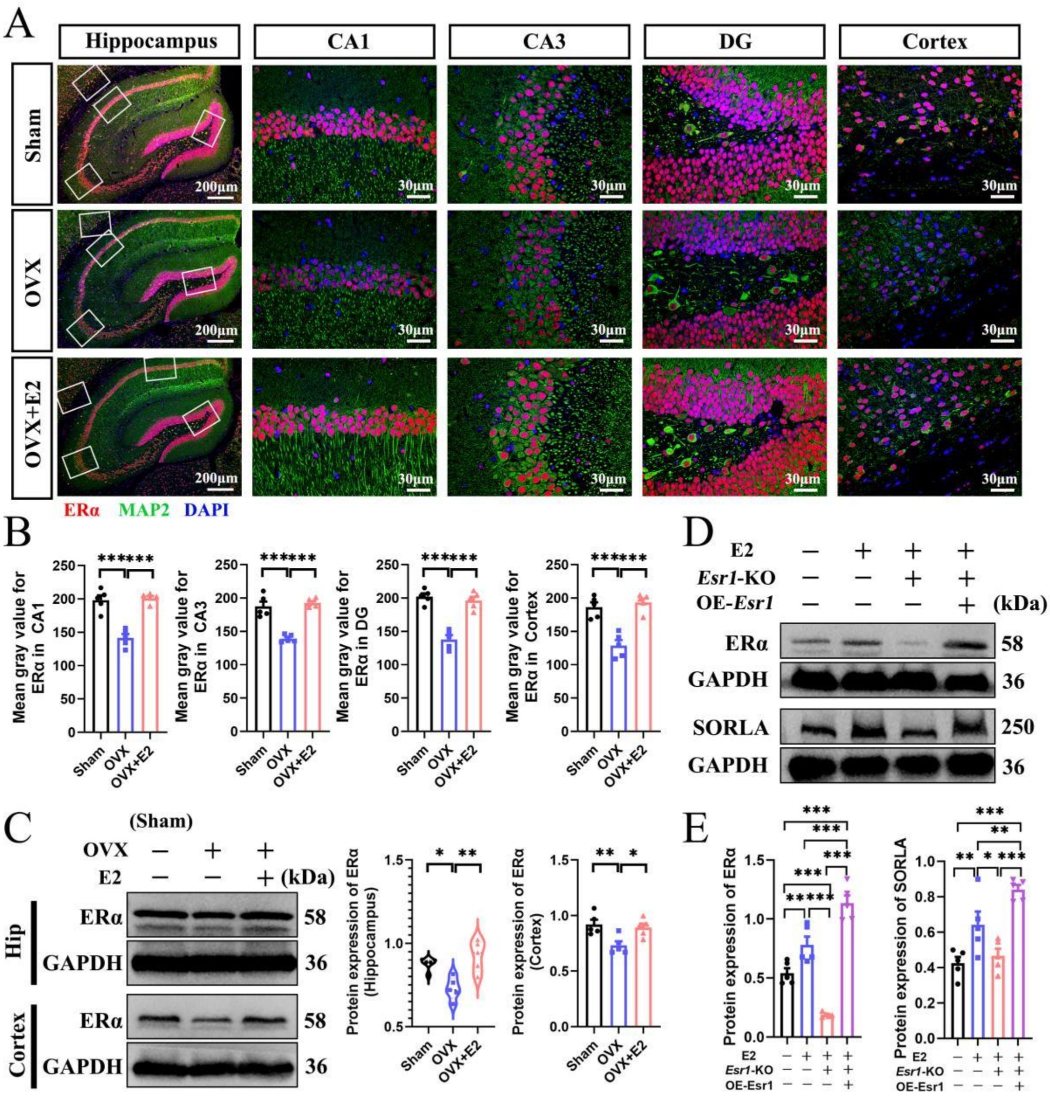

Fig. S4. Estradiol enhances SORLA expression via ERα signaling. (A, B) IHC for ERα expression in hippocampus and cerebral cortex. (C) WB for ERα expression in hippocampus and cerebral cortex. (D, E) WB for ERα and SORLA expression in HT22-APPswe cells, HT22-APPswe cells+E2, *Esr1*-KO HT22-APPswe

cells+OE-*Ctrl*+E2, and *Esr1*-KO HT22-APPswe cells+OE-*Sorl1*+E2. *P < 0.05, ***P < 0.001.

Fig.S5

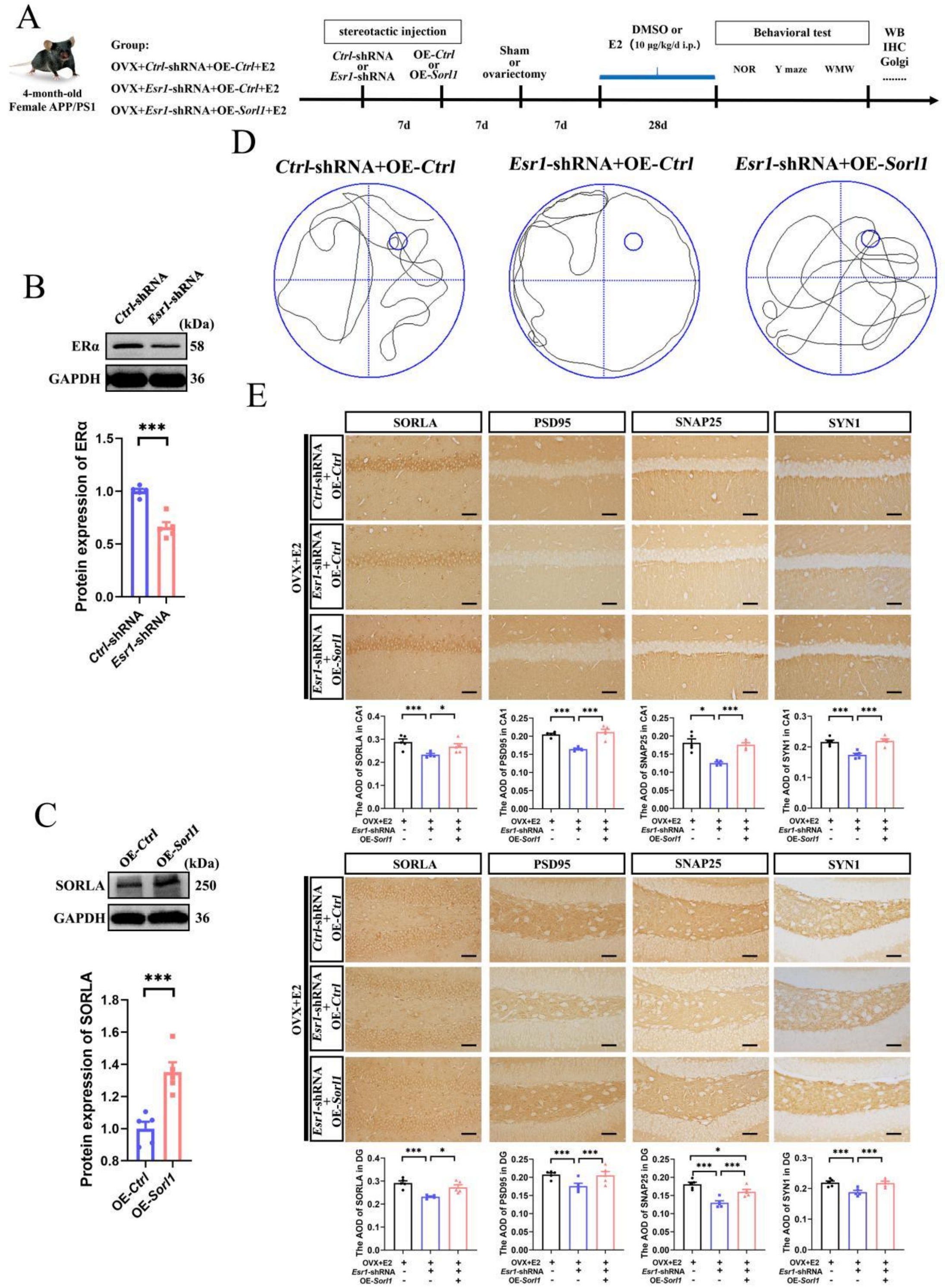

Fig. S5. The effect of estradiol mediated by ERα on improving synaptic damage in female APP/PS1-OVX mice is dependent on SORLA. (A) Experimental

procedures of ovariectomy, estradiol administration, virus injection and behavioral tests. (B) WB for ERα expression in APP/PS1-OVX mice treated with *Ctrl*-shRNA or *Esr1*-shRNA. (C) WB for SORLA expression in APP/PS1-OVX mice treated with OE-*Ctrl* or OE-*Sorl1*. (D) Track plots of MWM. (E) IHC for SORLA, PSD95, SNAP25, and SYN1 expression in the hippocampal CA1 and DG regions. OVX+*Ctrl*-shRNA+OE-*Ctrl*+E2, female ovariectomized APP/PS1 mice treated with *Ctrl*-shRNA, OE-*Ctrl*, and E2; OVX+*Esr1*-shRNA+OE-*Ctrl*+E2, female ovariectomized APP/PS1 mice treated with *Esr1*-shRNA, OE-*Ctrl*, and E2; OVX+*Esr1*-shRNA+OE-*Sorl1*+E2, female ovariectomized APP/PS1 mice treated with *Esr1*-shRNA, OE-*Sorl1*, and E2. **P* < 0.05, ****P* < 0.001.

Table S1. GO enrichment genes associated with Aβ metabolism

GO_ID	Biological Process Involving Amyloid-beta	Gene Names
GO:1904645	response to amyloid-beta	Gsk3β, Trem2, Epha4, Grm5, Cd36, Chrna7
GO:1904646	cellular response to amyloid-beta	Gsk3β, Trem2, Epha4, Grm5, Cd36
GO:0001540	amyloid-beta binding	Cd74, Trem2, Chrna7, Sorl1, Itga2, Cd36
GO:0150094	amyloid-beta clearance by cellular catabolic process	Cd36, Lrp4
GO:1902430	negative regulation of amyloid-beta formation	Chrna7, Sorl1, Ntrk2
GO:1902003	regulation of amyloid-beta formation	Epha4, Chrna7, Sorl1, Ntrk2
GO:1902992	negative regulation of amyloid precursor protein catabolic process	Chrna7, Sorl1, Ntrk2
GO:1902959	regulation of aspartic-type endopeptidase activity involved in amyloid precursor protein catabolic process	Sorl1, Epha4
GO:0034205	amyloid-beta formation	Epha4, Chrna7, Sorl1, Ntrk2
GO:1902991	regulation of amyloid precursor protein catabolic process	Epha4, Chrna7, Sorl1, Ntrk2
GO:0042982	amyloid precursor protein metabolic process	Ago2, Epha4, Chrna7, Sorl1, Ntrk2
GO:1902962	regulation of metalloendopeptidase activity involved in amyloid precursor protein catabolic process	Sorl1
GO:1902963	negative regulation of metalloendopeptidase activity involved in amyloid precursor protein catabolic process	Sorl1
GO:0097242	amyloid-beta clearance	Cd36, Lrp4, Trem2
GO:0050435	amyloid-beta metabolic process	Epha4, Chrna7, Sorl1, Ntrk2
GO:1902960	negative regulation of aspartic-type endopeptidase activity involved in amyloid precursor protein catabolic process	Sorl1
GO:0042987	amyloid precursor protein catabolic process	Epha4, Chrna7, Sorl1, Ntrk2
GO:1902004	positive regulation of amyloid-beta formation	Epha4, Chrna7
GO:1990000	amyloid fibril formation	Chrna7, Cd36
GO:1902993	positive regulation of amyloid precursor protein catabolic process	Epha4, Chrna7
GO:0042985	negative regulation of amyloid precursor protein biosynthetic process	Ago2
GO:1900223	positive regulation of amyloid-beta clearance	Trem2
GO:1902961	positive regulation of aspartic-type endopeptidase activity involved in amyloid precursor protein catabolic process	Epha4

GO:0042983	amyloid precursor protein biosynthetic process	Ago2
GO:0042984	regulation of amyloid precursor protein biosynthetic process	Ago2
GO:1905906	regulation of amyloid fibril formation	Chrna7
GO:1900221	regulation of amyloid-beta clearance	Trem2

Table S2. Antibodies used in the experiments

Antibody	Supplier	Catalog #	Application
PSD95	Proteintech	20665-1-AP	IHC (1:200); WB (1:1000)
SNAP25	Proteintech	14386-1-AP	IHC (1:200); WB (1:1000)
SYN1	Proteintech	20258-1-AP	IHC (1:200); WB (1:1000)
β-amyloid	Abcam	ab201060	IHC (1:200)
SORLA	Proteintech	22592-1-AP	IHC (1:200); WB (1:500); IF (1:200); Co-IP (10μg/ml); PLA (1:200)
APP	Proteintech	60342-1-Ig	WB (1:1000); Co-IP (10μg/ml); PLA (1:200); IF (1:200)
sAPPβ	BioLegend	813401	WB (1:2000)
ERα	Sigma-Aldrich	06-935	WB (1:1000); ChIP(4 μg)
EEA1	CST	3288	IF (1:200); WB (1:1000)
TGN38	CST	65969	IF (1:200)
Rab7	CST	9367	IF (1:200)
LAMP1	CST	99437	IF (1:200)
Normal Mouse IgG	Beyotime	P2179S-5	Co-IP (10μg/ml)
Normal Rabbit IgG	Beyotime	P2179S-6	Co-IP (10μg/ml); ChIP (4 μg)
GAPDH (mouse)	Proteintech	60004-1-Ig	WB (1:100000)
GAPDH (rabbit)	Proteintech	10494-1-AP	WB (1:10000)
HRP-conjugated Goat anti-mouse	Proteintech	SA00001-1	WB (1:100000)
HRP-conjugated Goat anti-rabbit	Proteintech	SA00001-2	WB (1:100000)
Alexa Fluor 488-labeled Donkey Anti-Rabbit antibody	Abcam	ab150061	IF (1:200)
Alexa Fluor 647-labeled Donkey Anti-Mouse antibody	Abcam	ab150107	IF (1:200)

Table S3. Primers used for PCR and ChIP

Genes	Species	Forward primer (5'-3')	Reverse primer (5'-3')
Trem2	mouse	CTGGTGGAGGTGCTGGAGGAC	AAGAATGGAGGTGGGTGGAAGG
Cd74	mouse	GCCAGGAAGAAGTCAGCCACATC	GTTACCGTTCTCGTCGCACTTGG
Itga2	mouse	CTCCTGCTGCGGCTGCTAATG	AGTTGCCTTGTGGGTTCGTAAGC
Epha4	mouse	GGAAGGAGGGTGGGAGGAAGTG	AGTCAGTTCGCAGCCAGTTGTTC
Lrp4	mouse	CCCTTCAGCACTACCCTCTCCTC	ACCAGTCTCCACAGTCGTCATCTC
Ago2	mouse	TTCCGACACCTGAAGAACACATACG	ACACGCTTGACTTCCGCATACAC
Grm5	mouse	CAGTCCGTGAGCAGTATGGCATTC	CCTAGTGTGATGTTGGGCAAGAGTG
GSK3β	mouse	GGCTGTGTGTTGGCTGAATTGTTG	TTTGCTCCCTTGTTGGTGTTCCTAG
Ntrk2	mouse	GGTGGCTGTGAAGACGCTGAAG	AATGTGCTCGTGCTGGAGGTTG
Cd36	mouse	GGTCTATCATCGCTGTGTTCGGATC	GCAAAGGCATTGGCTGGAAGAAC
Chrna7	mouse	GGGTCGTGTGTGGTCGTTTGG	CGGAAGCGGTTGGCGATGTAG
Sorl1(qRT-PCR)	mouse	CCAAACGCTATCTACATTGAGC	GCAAACATATACTTGTCGCGAA
Esr1	mouse	CTACTACCTGGAGAACGAGC	GCGTCGATTGTCAGAATTAGAC
APP	human	ACGATGAGGATGGTGATGAGGTAG	GTGGCAATGCTGGTGGTTCTC
GAPDH	mouse	GGTTGTCTCCTGCGACTTCA	TGGTCCAGGGTTTCTTACTCC

Sorl1 (ChIP)	mouse	CTGGTGGGAGCAGTTAAGTAT	TCTCTAAACCAATGGACGGCT
Sorl1 Region 1	mouse	GCCATTCCCATAGCCTACAGAT	CAGATATTTTGGTCCCAGTCATGC
Sorl1 Region 2	mouse	ATGACTGCTTCTTGTCATTTTGGTC	CTATAGGAGGTAAGGCCTGGCTA
Sorl1 Region 3	mouse	GCCATTCCCATAGCCTACAGAT	CTATAGGAGGTAAGGCCTGGCTA
Esr1 Region 1	mouse	CAGTTTCATTCCTGGGATTTGCTC	ATAATGGTAGCCAGAGGCATAGTC
Esr1 Region 2	mouse	AGGTCTAATTCTGACAATCGACGC	AACTGTACACAAAGAGGGACCAAG
Esr1 Region 3	mouse	CAGTTTCATTCCTGGGATTTGCTC	AACTGTACACAAAGAGGGACCAAG

Original Western Blots

1.The original Western blot results for Fig. 1

(E) PSD95

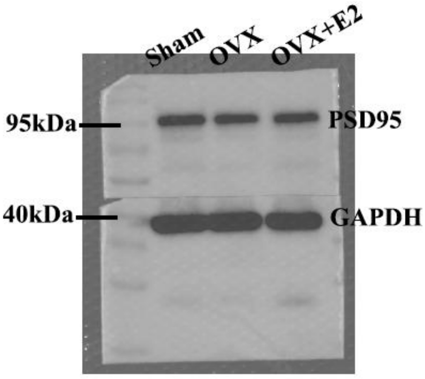

(E) SNAP25

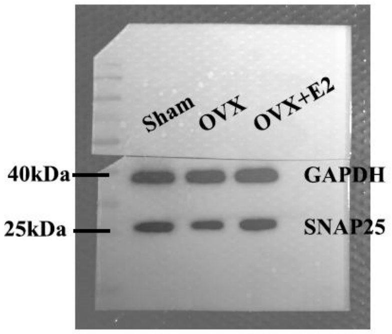

(E) SYN1

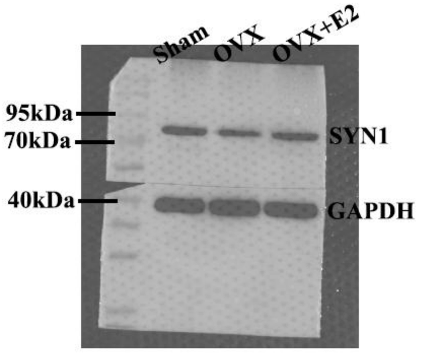

(E) APP

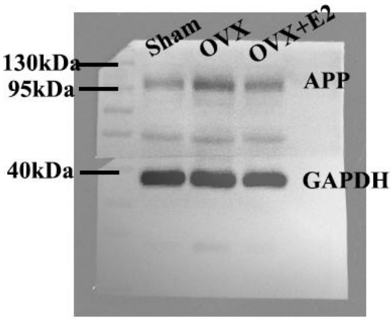

(E) sAPPβ

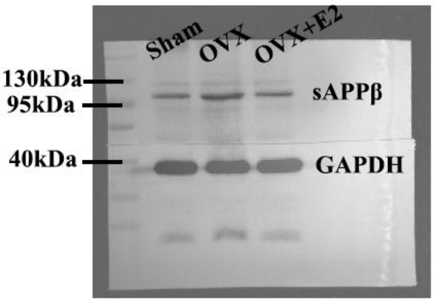

2.The original Western blot results for Fig. 2

(F) SORLA

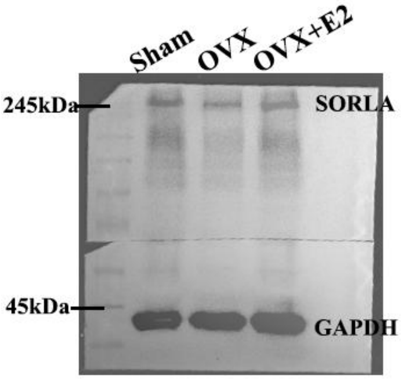

3.The original Western blot results for Fig. 3

(E) PSD95

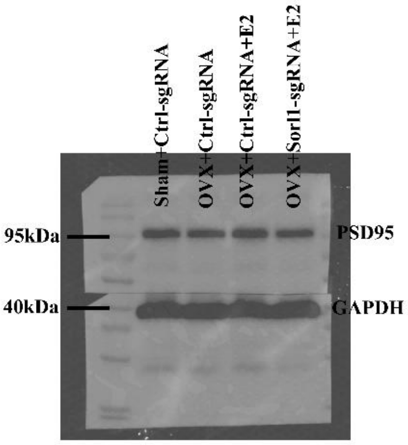

(E) SNAP25

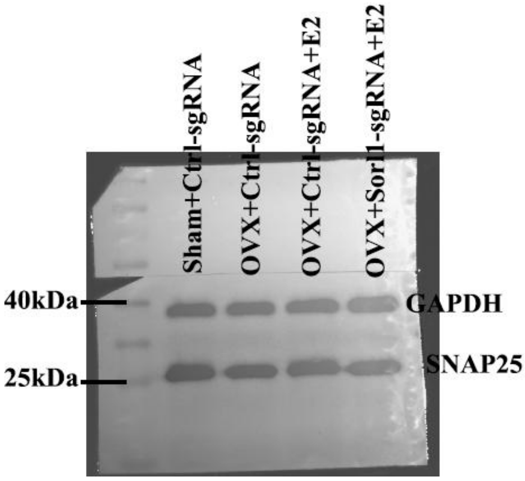

(E) SYN1

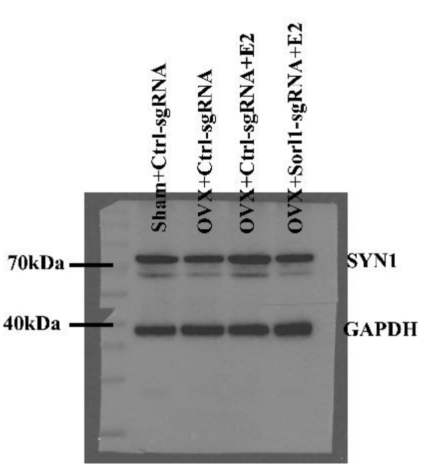

(E) APP

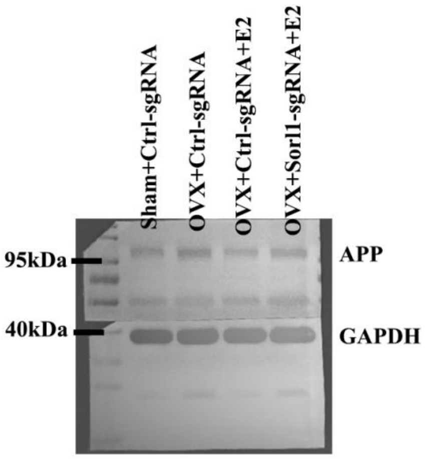

(E) sAPPβ

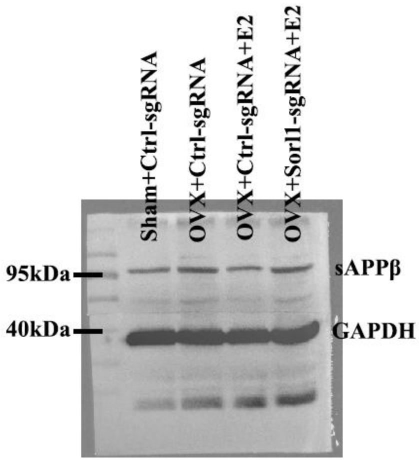

4.The original Western blot results for Fig. 4

(B) SORLA (APP/PS1 hippocampus)

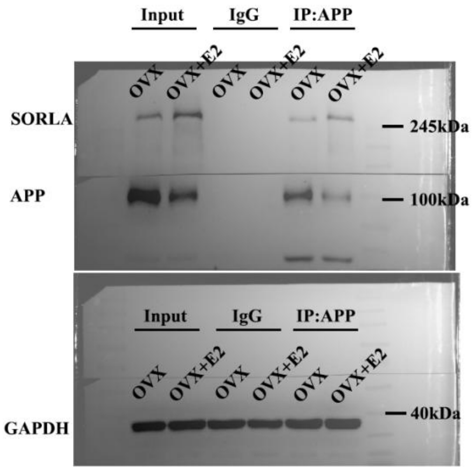

(C) SORLA (HT22-APPswe)

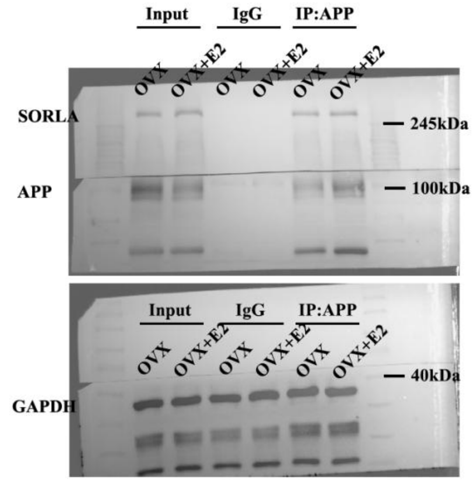

(F) SORLA (HT22-APPswe)

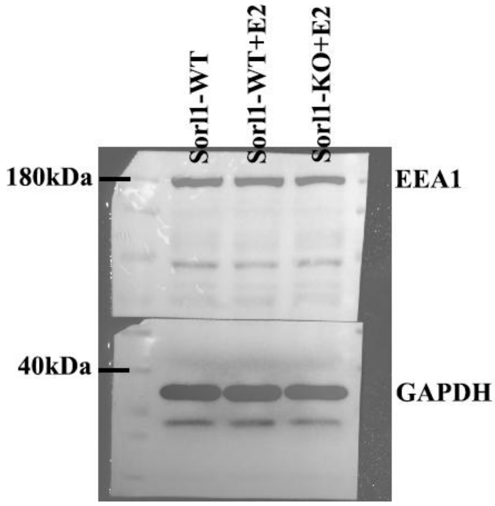

5.The original Western blot results for Fig. 5

(A) SORLA

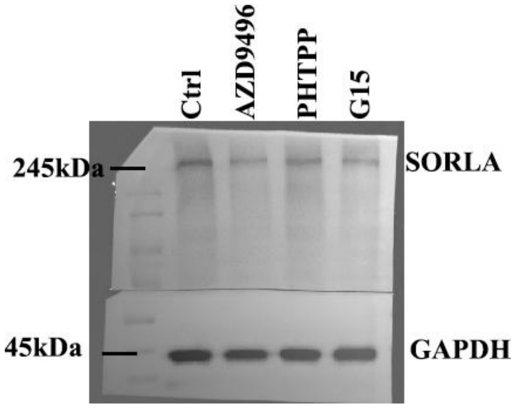

(G) SORLA

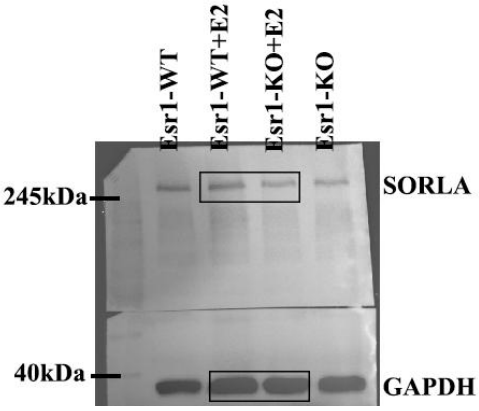

(G) APP

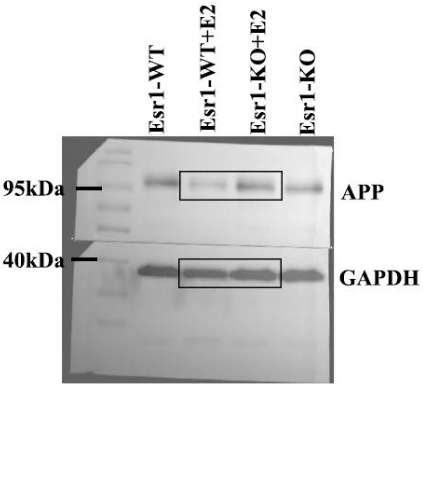

(G) sAPPβ

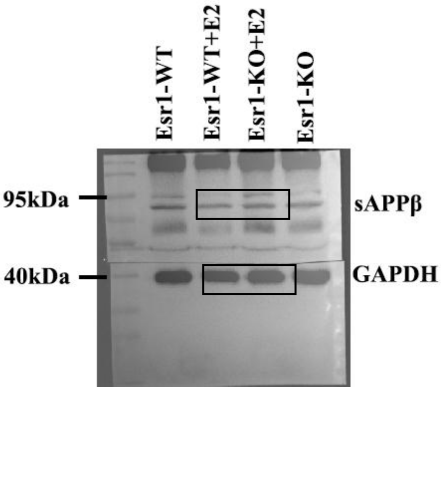

6.The original Western blot results for Fig. 6

(E) SORLA

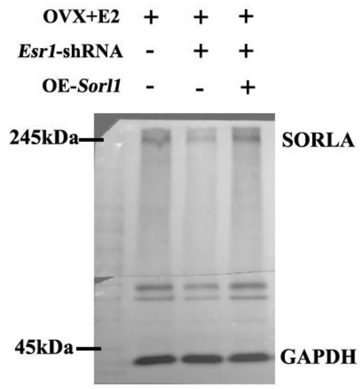

(E) PSD95

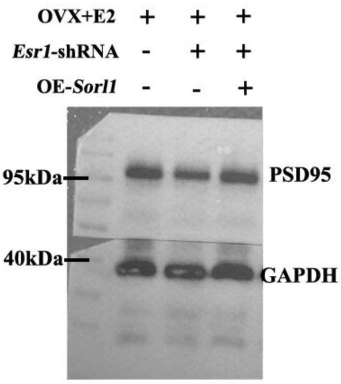

(E) SNAP25

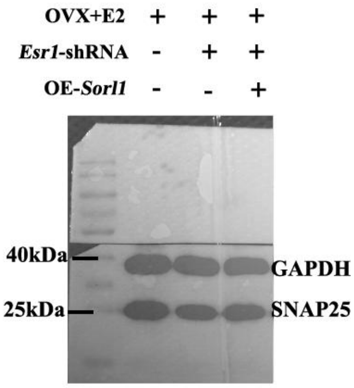

(E) SYN1

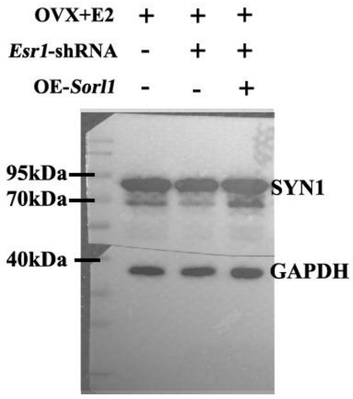

(E) APP

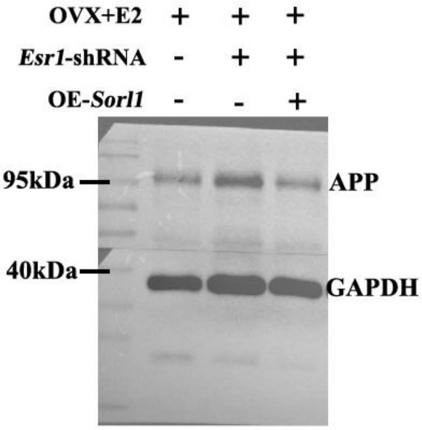

(E) sAPPβ

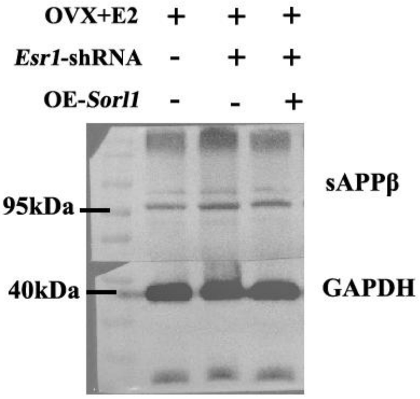

7.The original Western blot results for Fig. S2

(A) SORLA

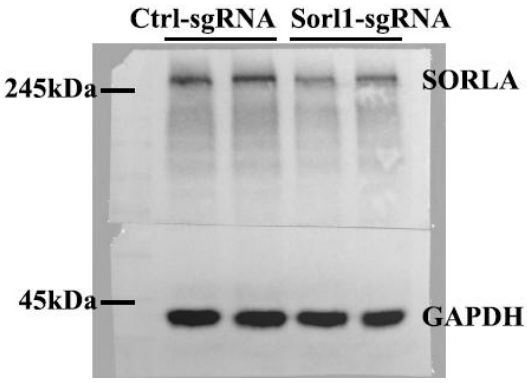

8.The original Western blot results for Fig. S3

(B) APP

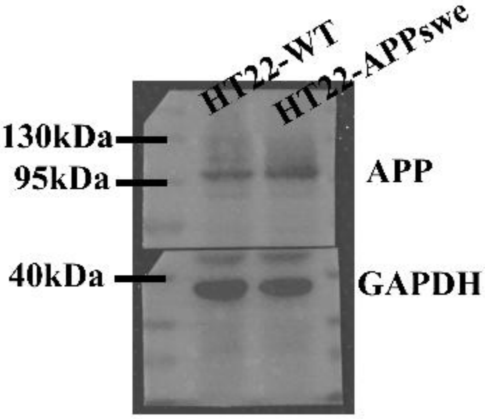

9.The original Western blot results for Fig. S4

(C) ERα (hippocampus)

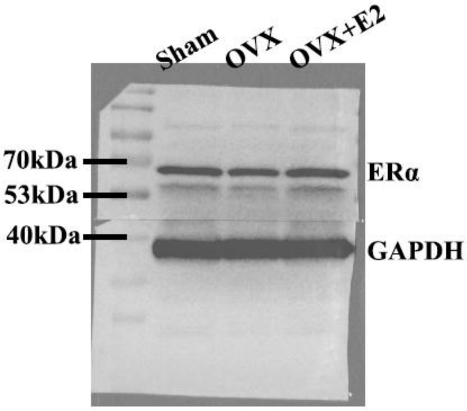

(C) ERα (cortex)

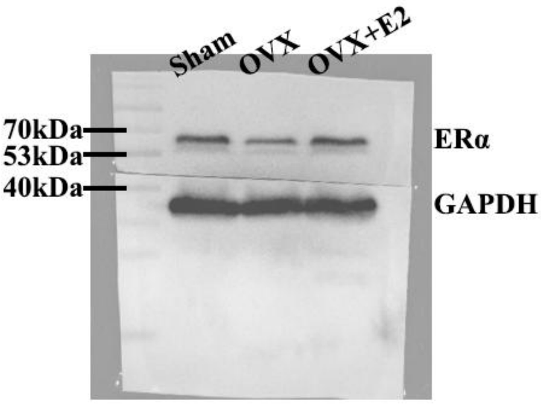

(D) ERα

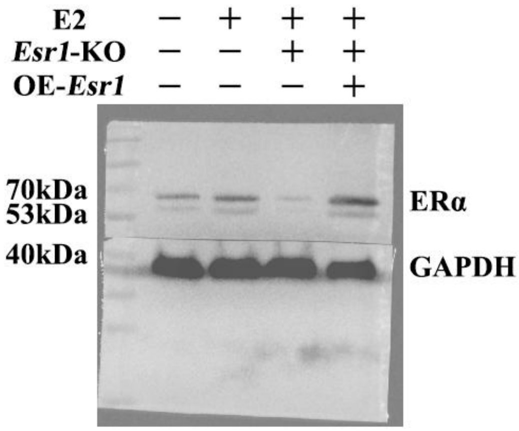

(D) SORLA

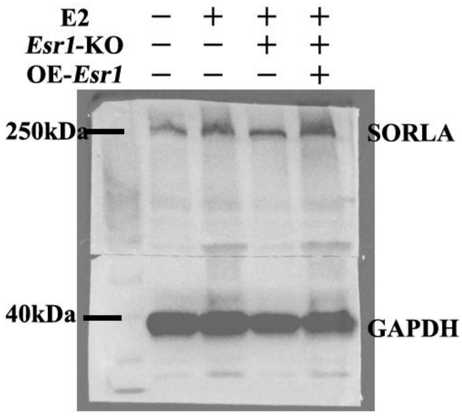

10.The original Western blot results for Fig. S5

(B) ERα

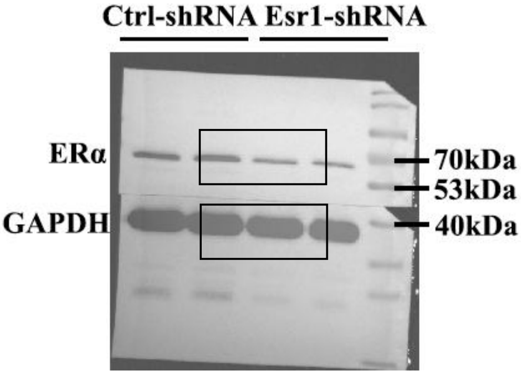

(C) SORLA

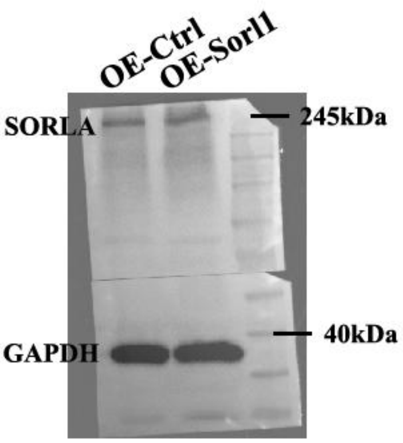

Supplement: Supplementary file 1 — Supplementary Material 1. [file 13195_2026_2027_MOESM1_ESM.pdf]
